# The Causal Association between Alcohol, Smoking, Coffee Consumption, and the Risk of Arthritis: A Meta-Analysis of Mendelian Randomization Studies

**DOI:** 10.3390/nu15235009

**Published:** 2023-12-04

**Authors:** Junxiang Wang, Binfei Zhang, Leixuan Peng, Jiachen Wang, Ke Xu, Peng Xu

**Affiliations:** 1 Department of Joint Surgery, Honghui Hospital, Xi’an Jiaotong University, Xi’an 710054, China; qw2712723867@stu.xjtu.edu.cn (J.W.); beephoe@stu.xjtu.edu.cn (B.Z.); wjc8128@stu.xjtu.edu.cn (J.W.); santxuke1986@126.com (K.X.); 2 The School of Medicine, Xi’an Jiaotong University, Xi’an 710049, China; 2196320198@stu.xjtu.edu.cn

**Keywords:** osteoarthritis, rheumatoid arthritis, mendelian randomization, alcohol, smoking, coffee

## Abstract

**Objective**: To evaluate the genetic causality between alcohol intake, smoking, coffee consumption, and arthritis. **Methods**: Mendelian randomization (MR) studies with alcohol, smoking, and coffee consumption behaviors as exposures, and osteoarthritis (OA) and rheumatoid arthritis (RA) as outcomes were retrieved from up to July 2023. Two researchers with relevant professional backgrounds independently assessed the quality and extracted data from the included studies. Meanwhile, we applied MR analyses of four lifestyle exposures and five arthritis outcomes (two for OA and three for RA) with gene-wide association study (GWAS) data that were different from the included studies, and the results were also included in the meta-analysis. Statistical analyses were performed using Stata 16.0 and R software version 4.3.1. **Results**: A total of 84 studies were assessed. Of these, 11 were selected for meta-analysis. As a whole, the included studies were considered to be at a low risk of bias and were of high quality. Results of the meta-analysis showed no significant genetic causality between alcohol intake and arthritis (odds ratio (OR): 1.02 (0.94–1.11)). Smoking and arthritis had a positive genetic causal association (OR: 1.44 (1.27–1.64)) with both OA (1.44 (1.22–1.71)) and RA (1.37 (1.26–1.50)). Coffee consumption and arthritis also had a positive genetic causal association (OR: 1.02 (1.01–1.03)). Results from the subgroup analysis showed a positive genetic causality between coffee consumption and both OA (OR: 1.02 (1.00–1.03)) and RA (OR: 1.56 (1.19–2.05)). **Conclusion**: There is positive genetic causality between smoking and coffee consumption and arthritis (OA and RA), while there is insufficient evidence for genetic causality between alcohol intake and arthritis.

## 1. Introduction

Arthritis is an inflammatory disease that occurs in the joints and causes structural damage and pain, with osteoarthritis (OA) and rheumatoid arthritis (RA) being the most common types of arthritis [1]. Osteoarthritis, primarily characterized by articular cartilage loss and subchondral bone changes, affects more than 500 million people worldwide and is one of the leading causes of pain and joint mobility impairment, resulting in significant disease and economic burdens [2,3]. RA is a systemic inflammatory autoimmune disease characterized by synovial inflammation that causes skeletal muscle pain, swelling, and stiffness [4]. The prevalence of RA is approximately 0.5%–1%. RA can affect other parts of the body, especially the vascular system and metabolism, in addition to the joints. Compared to the general population, people with RA are at a greater risk of serious infections, respiratory diseases, cardiovascular diseases, and death [4,5]. Epidemiological studies have shown that the number of people affected by OA increased by 48% from 1990 to 2019. At the same time, a downward trend in the incidence of RA has not been observed [2,6].

Lifestyle provides great influences on arthritis onset and progression, and such is the case for OA and RA [7,8]. Observational studies have shown a correlation between arthritis and three common habits, namely, smoking, alcohol intake, and coffee consumption [9,10,11,12,13]. Substantial efforts have been made to investigate the role of different lifestyles and behaviors in the development of arthritis. However, high-quality randomized controlled trials (RCTs) are limited due to research ethics considerations. For example, prolonged patient exposure to risk factors, such as smoking and alcohol intake, may lead to other forms of health hazards, which is contrary to research ethics.

Mendelian randomization (MR) is an epidemiological study method that uses genetic variants associated with potential risk factors as instrumental variables (IVs) to explore the genetic causality between exposure and outcome [14]. In MR, groups for natural exposure and controls are created by randomly allocating alleles to offspring in accordance with Mendel’s law. MR studies are recognized as having a high degree of similarity to RCTs [15]. Compared to traditional observational studies, the influence of confounding factors is reduced owing to the random assignment of genetic variants, and the genetic variants must occur before disease onset, which eliminates the risk of reverse causality bias [16]. Due to these positive study design characteristics and their high reliability, MR studies have become increasingly common.

In the past few years, the potential causal relationships between arthritis and alcohol, smoking, and coffee consumption have been examined using MR methods [17,18,19,20,21,22,23,24,25,26,27]. These studies varied in quality, design, instrumental variables, cohort, and causal effect estimates, leading to confusion. We conducted a systematic review of these studies to comprehensively assess the evidence for genetic causality between alcohol intake, smoking, and coffee consumption with arthritis. We also conducted MR analyses using a different GWAS from the included studies and meta-analyzed the results together.

## 2. Materials and Methods

### 2.1. Mendelian Randomization Analysis

#### 2.1.1. Data Sources

We obtained GWAS datasets from a study of 1.2 million people with alcoholic drinks per week and cigarette smoking initiation [28]. Meanwhile, we used the IEU OpenGWAS project (gwas.mrcieu.ac.uk, accessed on 7 September 2023) to obtain another GWAS dataset of alcohol intake frequency (containing 462,346 individuals) and coffee consumption (containing 428,860 individuals) from the UK Biobank.

For outcomes, we obtained the GWAS dataset for OA from a genome-wide meta-analysis of up to 826,690 individuals (177,517 patients) [29]. The summary-level data for hip OA (HOA) included 36,445 patients with HOA and 316,943 controls, and the summary-level data for knee OA (KOA) included 62,497 patients and 333,557 controls. The dataset for RA was obtained from FinnGen (www.finngen.fi/en, accessed on 7 September 2023), and the three outcomes of seropositive rheumatoid arthritis, seronegative rheumatoid arthritis, and rheumatoid arthritis meeting the definition of M13 (ICD-10 International Statistical Classification of Diseases) were selected.

#### 2.1.2. Selection of Instrumental Variables

To ensure that the IVs met the three basic assumptions of MR with sufficient validity, we used stringent conditions to screen single nucleotide polymorphisms (SNPs). First, SNPs significantly associated with exposure at the genome-wide level *p* < 5 × 10^−8^ were selected and the SNPs with the linkage disequilibrium effect were removed (r^2^ < 0.001, window size = 10,000 kb). We then screened and excluded SNPs (*p* < 5 × 10^−8^) associated with confounders (body mass index was removed when OA was used as the outcome) using the PhenoScanner database (http://www.phenoscaner.medschl.cam.ac.uk/, accessed on 8 September 2023). In addition, to further exclude possible associations between IVs and outcome, we excluded SNPs associated with outcome (*p* < 5 × 10^−8^). We also excluded palindromic variants that could not be oriented (i.e., C/G or A/T). F statistics were also computed, and the selected IVs all satisfied F > 10 to avoid possible bias caused by weak instrumental variables [30].

#### 2.1.3. Statistical Analysis Methods

The inverse-variance weighted (IVW) method was used for primary analysis, and the weighted model was used for secondary analyses. The IVW method assumes that all IVs are valid, and provides the most accurate estimates based on the calculation of Wald ratios [31]. The weighted median method is an improvement over the simple median method and provides consistent estimates when at least 50% of the instrumental variables are valid [32]. Multiple sensitivity analyses were used to assess the estimates of the causal associations between exposure and outcomes. The I^2^ index and Cochran’s Q statistic were calculated using IVW analyses to assess heterogeneity. Potential horizontal pleiotropy between IVs was demonstrated by the intercept of the MR-Egger method [33]. MR-PRESSO was also applied, which detects and corrects for horizontal pleiotropy by removing outliers at *p* < 0.05 and returns the corrected causal effects [34].

All statistical data analyses were performed using the TwoSampleMR package and the MendelR package in R software version 4.3.1.

### 2.2. Study Search and Inclusion Criteria

This study is based on the 2020 update of the (PRISMA-P) guidelines and is registered with PROSPERO (CRD42022375445). We searched the MEDLINE, EMBASE, and Cochrane Library databases using the following key words: alcohol, drinking, smoking, cigarette, coffee, Mendelian randomization, and osteoarthritis, rheumatoid arthritis, arthritis. The search period was from the time that each database was created to July 2023. The search process was not qualified otherwise.

The inclusion criteria for the retrieved studies were as follows:

1. Any studies using the MR method to investigate causality between risk factors and osteoarthritis/rheumatoid arthritis or related phenotypes.
2. Any studies on gender, age, cohort, and race.
3. Any studies that include MR as part of their analysis.

The exclusion criteria were as follows:

1. Any case reports, narrative reviews, or other non-research-based studies.
2. Any studies that did not include alcohol/smoking/coffee consumption in the exposures or OA/RA in the outcomes.
3. Any studies of non-European origin cohorts.
4. Any studies for which the complete English manuscript is not available.

### 2.3. Data Extraction and Quality Assessment

Two trained researchers with relevant professional backgrounds independently read titles and abstracts of the literature. By reading the full text of all articles that met the inclusion criteria, researchers extracted information about specific methods and measures from the study design. We extracted data from the included literature and assessed the study quality based on the Guidelines for Strengthening the Reporting of Mendelian Randomization Studies (STROBE-MR) and its expansion statement, while referring to the Mendelian Randomization Reading Guide [35,36,37].

### 2.4. Meta-Analysis

Meta-analyses were performed using the “metan” command in Stata 16.0 to obtain pooled effect estimates. The primary outcome was the risk of diagnosis and development of arthritis, and subgroup analyses were performed for OA and RA. Since alcohol, smoking, and coffee consumption affect all parts of the body, OA and RA in different parts of the body or different subtypes are considered the same outcome to explore the overall impact on the disease. We used ORs and 95% confidence intervals to report pooled estimates of the causal effect between lifestyle and arthritis, and a two-sided *p* value < 0.05 under a random-effects model was considered statistically significant. Inverse variance weighted (IVW) estimates were used as the primary analysis and the results of the weighted median method (WM) were used as a sensitivity analysis.

## 3. Results

### 3.1. Mendelian Randomization Analysis

**Figure 1:**
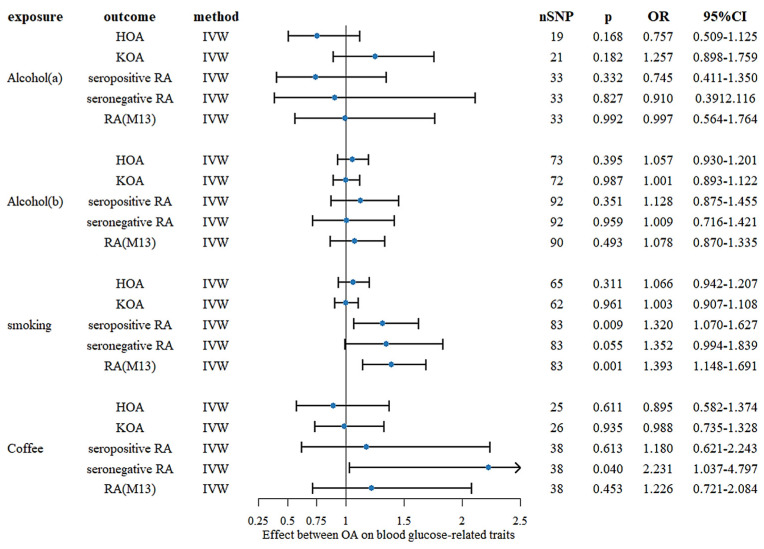
Forest plot of genetic causality between lifestyle and different arthritis outcomes assessed by the IVW MR method. Alcohol(a): Alcoholic drinks per week; Alcohol(b): Alcohol intake frequency; SNP: single nucleotide polymorphisms; SNP: single nucleotide polymorphisms; p: *p* value of the causal estimate; OR = odds ratio; CI, confidence interval.

The results of the MR analyses of lifestyle on OA and RA are displayed in Figure 1 and in the Supplementary Materials. The analyses indicated a lack of association between a genetic predisposition to higher alcohol intake and OA or RA outcomes. When smoking was used as an exposure, positive genetic causality was found for seropositive RA and RA meeting the definition of M13. ORs were 1.32 (1.07–1.63) and 1.39 (1.15–1.69). The analysis also revealed a positive genetic causality between coffee and seronegative RA with an OR of 2.23 (1.04–4.80). The MR-Egger test for all analyses suggested no pleiotropy (*p* > 0.05), as well as no outliers in the MR-PRESSO method. Detailed results of the analyses and sensitivity analyses are available in the Supplementary Materials.

### 3.2. Study Characteristics and Literature Inclusion

**Figure 2:**
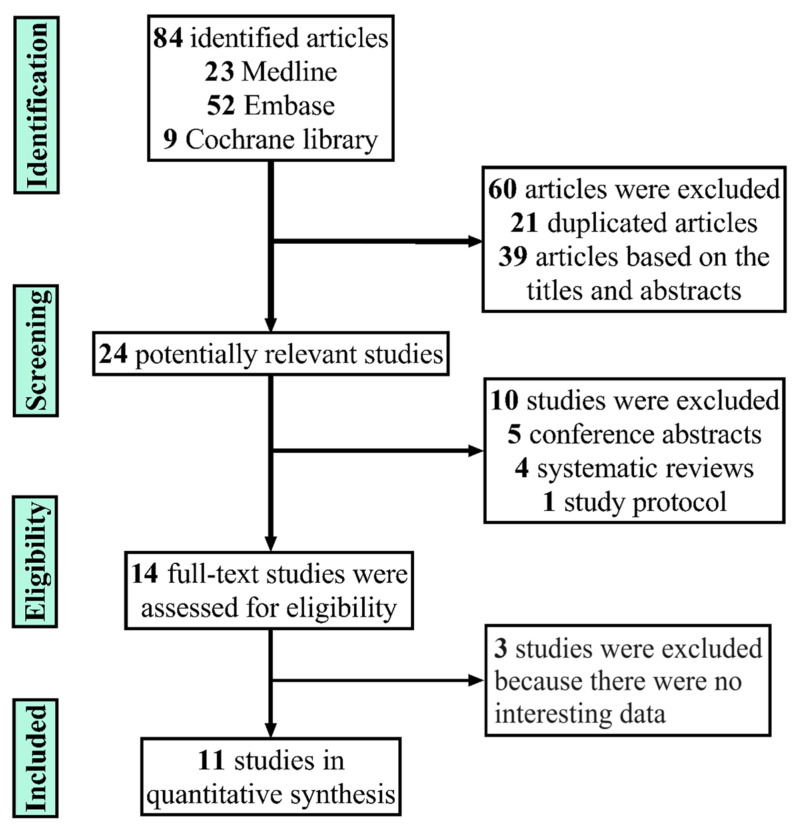
Flowchart of the studies included in the meta-analysis.

From the databases Medline, Embase, and the Cochrane Library, 23, 52, and 9 studies, were included, respectively. After reading the abstracts of the literature titles by the evaluators and excluding other apparently irrelevant studies, 14 studies were initially included. After reading the full text and excluding three studies that did not contain the index of interest, 11 studies were finally included. Figure 2 shows the study flowchart.

All 11 studies were in the English language and conducted by applying MR to alcohol intake, smoking, and coffee consumption as exposures, with OA and RA as the primary outcomes. The studies were published between 2018 and 2022. The included populations were of European origin, and the UK Biobank was the most used study cohort. Study characteristics of all the 11 included literature are shown in Appendix A, Table A1. We comprehensively assessed and assigned scores to all included studies based on the guidelines in up to 14 areas to determine the quality of their studies. The risk of bias in the literature was considered low after converting the total scores of the 14 assessed aspects into percentages if the score was >85% (Appendix A, Table A2) [17,18,19,20,21,22,23,24,25,26,27].

### 3.3. Results of Meta-Analysis

To examine the causal relationship between lifestyle and arthritis, a total of two studies related to alcohol intake [17,18], five studies related to smoking [19,20,21,22,23], and four studies related to coffee consumption [24,25,26,27] were included in the meta-analysis along with the results of our MR analysis.

#### 3.3.1. Alcohol Intake

**Figure 3:**
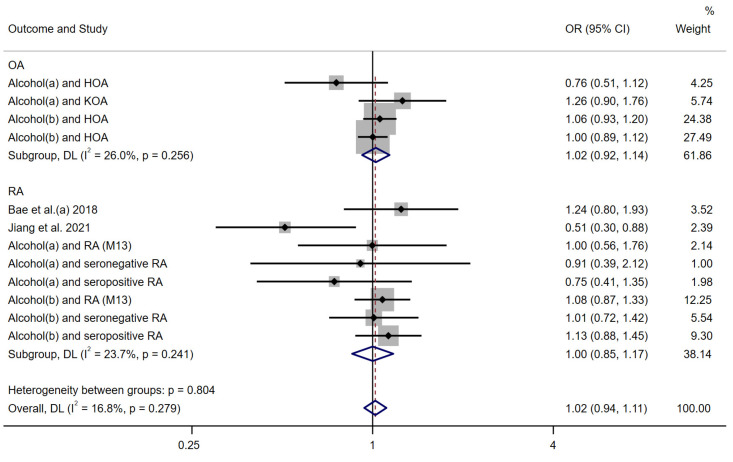
Forest plot of the IVW MR method to assess the genetic causal relationship between alcohol intake and RA. Alcohol(a): Alcoholic drinks per week; Alcohol(b): Alcohol intake frequency; OA: Osteoarthritis; RA: Rheumatoid arthritis; OR: Odds ratio; CI: Confidence interval; P: significance *p*-value; DL: DerSimonian and Laird approach [17,18].

We performed a meta-analysis using a random-effects model on the inverse variance weighted method estimates of the two included studies and our 10 MR analyses. The results showed that alcohol intake was positively associated with arthritis overall (OR: 1.02 (0.94–1.11)), but not statistically significant (Figure 3). Subgroup analyses divided into two subgroups, OA and RA, showed that the genetic predisposition between alcohol with OA and RA was also not significant (ORs: OA: 1.02 (0.92–1.14]); RA: 1.00 (0.85–1.17)).

**Figure 4:**
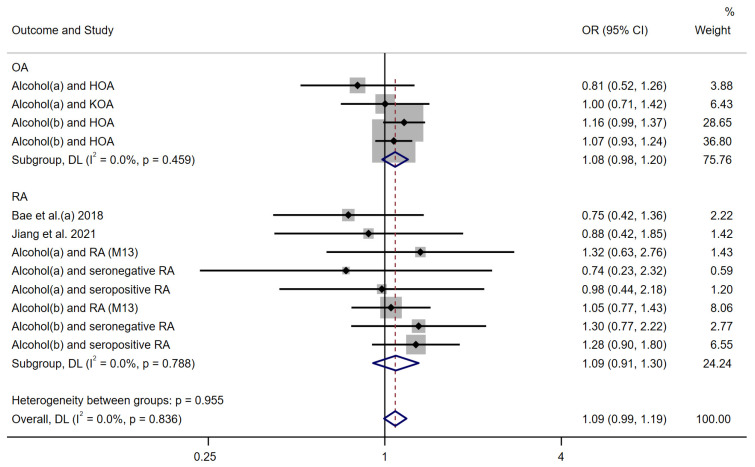
Forest plot of WM MR method to assess the genetic causality of alcohol intake and RA. Alcohol(a): Alcoholic drinks per week; Alcohol(b): Alcohol intake frequency; OA: Osteoarthritis; RA: Rheumatoid arthritis; OR: Odds ratio; CI: Confidence interval; P: significance *p*-value; DL: DerSimonian and Laird approach [17,18].

We then performed a meta-analysis of the estimates obtained using the weighted median method as a sensitivity analysis to assess the robustness of the IVW analysis. The combined results of the weighted median method under a random-effects model were consistent with previous results, with insufficient evidence of genetic causality between alcohol intake and arthritis overall (OR: 1.09 (0.99–1.19)). Subgroup analyses also reported insufficient evidence to support a genetic causality between alcohol and OA or RA (ORs: OA: 1.08 (0.98–1.20), RA: 1.09 (0.91–1.30)) (Figure 4).

#### 3.3.2. Smoking Behavior

**Figure 5:**
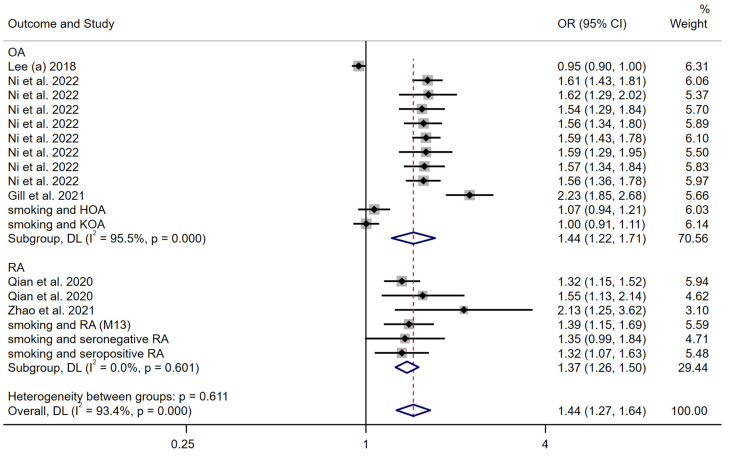
Forest plot of the IVW MR method to assess the genetic causality of smoking behavior and all arthritis outcomes. OA: Osteoarthritis; RA: Rheumatoid arthritis; OR: Odds ratio; CI: Confidence interval; P: significance *p*-value; DL: DerSimonian and Laird approach [19,20,21,22,23].

We performed a meta-analysis of the estimates of genetic causal effects between smoking behavior and arthritis, with outcomes obtained by the inverse variance weighted method using a random-effects model. The results revealed a positive causal association between smoking behavior and arthritis (OR: 1.44 (1.27–1.64)) (Figure 5). A subgroup analysis was performed by dividing the OA and RA subgroups. The results of the analysis indicated a positive genetic causality between smoking and both OA and RA, with an OR of 1.44 (1.22–1.71) and 1.37 (1.26–1.50), respectively. The strength of the genetic causal relationship of smoking for OA and RA are roughly equivalent.

**Figure 6:**
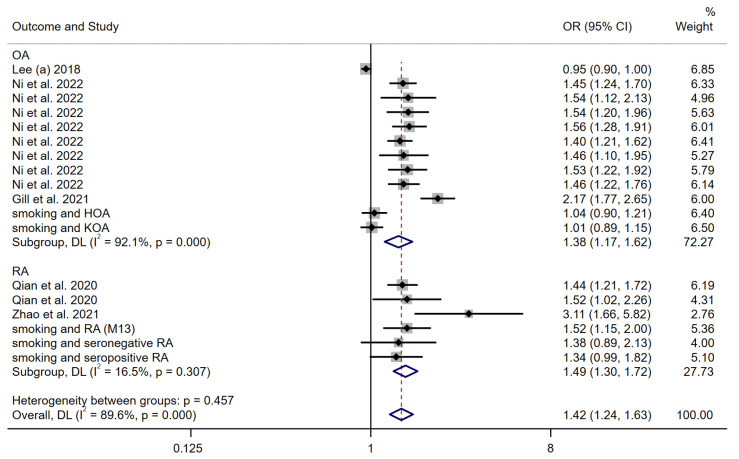
Forest plot of the WM MR method to assess the genetic causality of smoking behavior and all arthritis outcomes. OA: Osteoarthritis; RA: Rheumatoid arthritis; OR: Odds ratio; CI: Confidence interval; P: significance *p*-value; DL: DerSimonian and Laird approach [19,20,21,22,23].

As a sensitivity analysis, a meta-analysis of the genetic causal effect estimates of smoking behavior for all arthritis outcomes obtained using the weighted median method was performed. The results of the random-effects model remained consistent with previous studies, reporting a positive genetic causality between smoking behavior and arthritis (OR: 1.42 (1.24–1.63)) (Figure 6). The subgroup analysis confirmed stability of results, with ORs of 1.38 (1.17–1.62) and 1.49 (1.30–1.72), for OA and RA, respectively.

#### 3.3.3. Coffee Consumption

**Figure 7:**
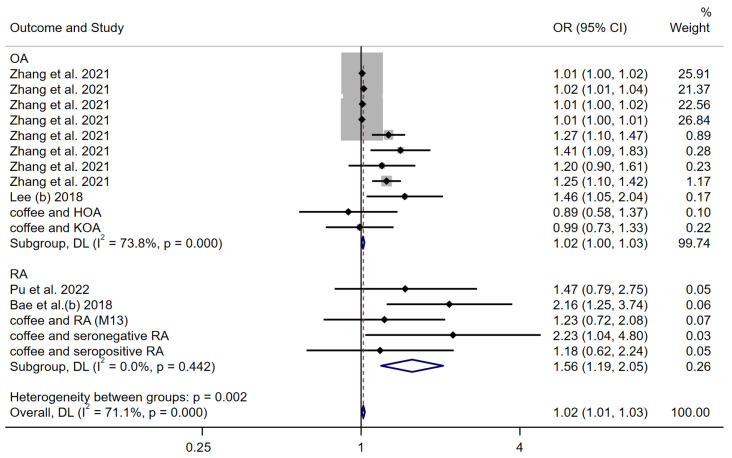
Forest plot of the IVW MR method to assess the genetic causality of coffee consumption and all arthritis outcomes. OA: Osteoarthritis; RA: Rheumatoid arthritis; OR: Odds ratio; CI: Confidence interval; P: significance *p*-value; DL: DerSimonian and Laird approach [24,25,26,27].

A meta-analysis was performed on the estimates of the genetic causal effects between coffee consumption and arthritis with outcomes obtained by the inverse variance weighted method using a random-effects model. The results revealed a positive causal association between coffee consumption and arthritis (OR: 1.02 (1.01–1.03)) (Figure 7). Divided into two groups (OA and RA), the subgroup analysis reported a positive genetic causality between coffee consumption and both OA (OR: 1.02 (1.00–1.03)) and RA (OR: 1.56 (1.19–2.05)). The genetic causal relationship between coffee consumption and RA was more significant than that between coffee consumption and OA.

**Figure 8:**
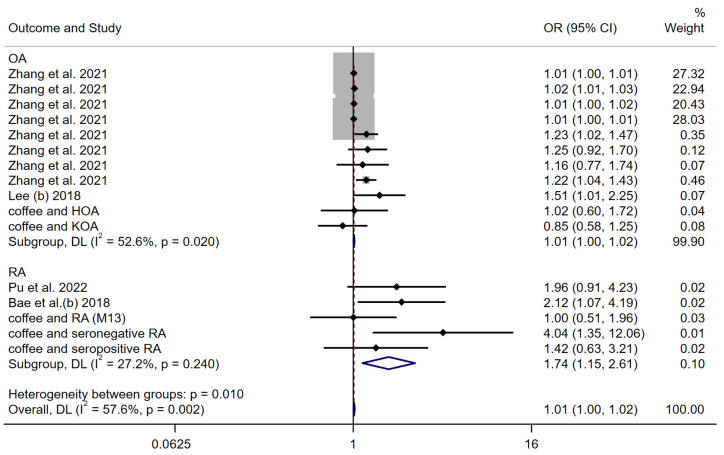
Forest plot of the WM MR method to assess the genetic causality of coffee consumption and all arthritis outcomes. OA: Osteoarthritis; RA: Rheumatoid arthritis; OR: Odds ratio; CI: Confidence interval; P: significance *p*-value; DL: DerSimonian and Laird approach [24,25,26,27].

As a sensitivity analysis, a meta-analysis of the genetic causal effect estimates of coffee consumption for all arthritis outcomes obtained using the weighted median method was performed. The results of the random effects model remained consistent with the findings of previous studies, and suggested a positive genetic causality between coffee consumption and arthritis (OR: 1.01 (1.00–1.02)) (Figure 8). The subgroup analysis also showed the stability of the results, with ORs of 1.01 (1.00–1.02) and 1.74 (1.15–2.61) for OA and RA, respectively.

## 4. Discussion

This study assessed the genetic causality between lifestyle and arthritis by applying MR methods and a meta-analysis of past MR studies, leading to the following findings: there was a significant positive causal relationship between smoking behavior and coffee consumption and arthritis (OA and RA). The strength of the genetic causal relationship of smoking for OA and RA were roughly equivalent, and the genetic causal relationship between coffee consumption and RA was more significant than that between coffee consumption and OA. There is insufficient evidence of a genetic causal relationship between alcohol intake and arthritis. These findings are consistent with the vast majority of qualitative analyses of alcohol, smoking, and coffee consumption. Overall, the included studies and this meta-analysis were considered to be of high quality.

### 4.1. Alcohol Intake

For alcohol intake and arthritis, the results of our meta-analysis showed that overall, there was insufficient evidence of genetic causality between them. There are many studies with differing opinions about alcohol and OA. It has been reported that alcohol consumption is negatively associated with the prevalence of osteoarthritis of the knee in the Korean population [38]. Another case-control study found that beer consumption appeared to be a risk factor for osteoarthritis of the knee and hip, while wine consumption was negatively associated with osteoarthritis of the knee [39]. A new study has found a positive correlation between alcohol consumption and the risk of hip osteoarthritis in women [40]. A meta-analysis by To, K. et al. that included 29 studies and 25,192 subjects with OA found that the negative association between alcohol and OA disappeared after adjusting for covariates, suggesting that the idea that alcohol prevents OA is unreliable [41].

Previous studies have also reported an association between alcohol intake and RA. A research study using data from the UK biobank showed a protective effect of alcohol on RA, with weekly or daily drinking associated with a reduced risk of RA compared with less than three drinks per month, and similar results were obtained in a cohort study of Swedish women [42,43]. In contrast, in an observational study of 16,762 individuals, Baker et al. showed no protective effect of alcohol consumption after excluding the confounding factors [44]. Another prospective study in a Chinese cohort discovered that drinking alcohol increased the risk of women developing RA [45].

As one of the most common lifestyles, alcohol intake affects the primary skeletal–muscular system as well as other systems of the body, and this compounding effect may be the reason for its unclear relationship with arthritis. For example, alcohol is associated with obesity, and increased joint loading is a risk factor for OA [46]. Alcohol consumption is associated with decreased muscle strength, which may also have a potential impact on the development of OA [47]. Different alcoholic beverages also have different effects, e.g., resveratrol in wine has shown protective effects on cartilage in in vitro experiments [48]. RA is a complex multifactorial disease. The reaction in the body’s immune system, such as antigen presentation, T-cell activation capacity of the APC, and maturation and proliferation of B cells, after alcohol intake, may be the potential mechanism of action [49]. Both acute and chronic alcohol consumption affects the gut microbiome, which can also have an impact on autoimmune diseases [50,51]. Different amounts of alcohol consumption also led to different results: moderate drinking caused a decrease in inflammatory markers compared to non-drinkers, while heavy drinking led to an increase in inflammatory markers [52].

### 4.2. Smoking Behavior

The results of our meta-analysis support a positive genetic causality between smoking behavior and arthritis, consistent with almost all the results included, and differing only from Lee’s study [19]. Previous studies on the connection between smoking and OA have been contentious. Data from an eight-year quality of life study in KOA patients showed that smoking was associated with a rapid deterioration in quality of life [53]. Another study showed that smoking was positively associated with serum high-sensitivity C-reactive protein (hsCRP) level for patients with early OA evident on imaging. In animal experiments using mice as a model, mice exposed to secondhand smoke exhibited more severe OA than the controls [54]. All of these studies provide evidence that smoking may be an independent risk factor for OA, which is consistent with our results, but at the same time, some scholars have reported different views. A meta-analysis found that smoking was negatively connected with the risk of knee OA, and this was more pronounced in men [10]. Two other studies showed that smoking is connected with a reduced risk of joint replacement but increases the incidence of postoperative complications and revision [55,56].

Smoking has been recognized as a relatively clear risk factor for RA development [57]. A prospective female cohort study in France found that passive smoking during childhood and/or adulthood increases the risk of RA in women [58,59]. A prospective 36-year follow-up of early smoking behavior in patients with RA showed that heavy smoking after RA diagnosis significantly increased mortality, whereas sustained smoking cessation over four years reduced the risk of death [60]. A cross-sectional study of US veterans showed that smoking was associated with elevated levels of pro-inflammatory cytokines and increased disease activity in patients with anti-CCP2-positive RA [61].

Cigarette smoke contains thousands of substances, including nicotine, polycyclic aromatic hydrocarbons, and nitric oxide, which can affect arthritis through various pathways. Chen et al. found that cigarette smoke extracts mediate cartilage destruction by increasing oxidative-stress-induced cell death [62]. Another review showed a dose-dependent relationship between nicotine and articular cartilage formation and osteogenesis, with lower doses generally having positive effects and higher doses having negative effects in different in vitro models [63]. This may partly explain the controversy over whether smoking is a risk factor for OA in observational studies. Moreover, anti-citrullinated cyclic peptide/protein antibody (ACPA) and rheumatoid factor (RF) are the two most prominent autoantibodies in patients with RA, and smoking is connected to an increase in ACPA and the development of joint symptoms [64]. Animal studies have found that smoking leads to increased arthritis and Th17 cells, and that these alternations are dependent on the transcription factor aryl hydrocarbon receptor (AHR) [65].

### 4.3. Coffee Consumption

For the results of a positive causal association between coffee consumption and arthritis, our meta-analysis was consistent with the results of several studies included in the systematic review [24,25,26,27]. Previous studies have not extensively reported on coffee consumption as a potential risk factor for OA, suggesting that the effect of coffee consumption on the development of OA may not be strong, which is reflected in our results. A national cross-sectional study found that an increase in the incidence of men’s knee OA was linked to daily coffee consumption of more than seven cups [13]. The effect of coffee consumption on RA is clearer than that on OA, and several studies have shown that coffee consumption is connected to the risk of RA and the degree of severity of the comorbidities [66,67,68]. In vitro studies have found that caffeine competitively inhibits the adenosine receptor A2a and promotes the production of IFN-γ in Th1 cells of patients with RA [69]. The cohort study also discovered that the frequency of RF positivity was positively correlated with the number of cups of coffee consumed each day, which may be another mechanism by which coffee may affect RA [70].

### 4.4. Strengths and Limitations

This is the first systematic review and meta-analysis of Mendelian Randomization studies on the causal relationship between arthritis and alcohol intake, smoking, and coffee consumption, to our knowledge. Owing to the increasing prevalence of MR studies, a pooled analysis of heterogeneous studies of the same outcome is necessary and meaningful.

This study has a number of strengths. Compared to traditional epidemiological studies, using the MR method can explore causality at the genetic level and can largely avoid bias caused by confounders and reverse causality, with highly reliable results. MR is a GWAS-based method, and the strength of evidence for outcomes is related to the sample size of GWAS participants. Our study meta-analyzed studies that used different GWAS in exposure or outcome, expanding the study cohort and sample size. We assessed the study quality in various aspects by referring to the STROBE-MR guidelines and MR reading guidelines. The results showed that the quality of the included literature, from the study design to the discussion of the results, was high and the risk of bias was low. This guarantees the robustness of the data and the results of this study.

For the meta-analysis, considering the heterogeneity across the literature, we applied a random-effects model for the analysis, and the results were still significant under conservative estimation. We estimated the results of the WM method as a sensitivity analysis in addition to the pooled IVW results to verify the robustness of the combined effects of the meta-analysis. The sensitivity analysis showed high agreement with the results of the main analysis, suggesting reliable results. Additionally, we performed a stratified analysis of two different types of arthritis, OA and RA, to assess the impact of a single lifestyle on specific outcomes.

This study has some limitations. The GWAS dataset was selected from populations of European ancestry, which avoids the effects of population stratification, but also means that more caution should be exercised when applying the findings to other ethnic groups. The GWAS datasets used in some of the earlier published studies included in the systematic review may not be the most recent and comprehensive, which may have affected the accuracy of the results. The absence of GWAS data on different age groups and sexes rendered stratified analyses of the individual-level characteristics challenging. Some sex-specific phenotypic differences may not be distinguishable because of this. As an emerging epidemiological method, the small number of included studies makes it difficult to assess publication bias for individual exposure factors, which can affect the reliability of results.

### 4.5. Clinical Implications

The influence of lifestyle as a part of the external environment on the development of arthritis is unquestionable [71]. However, whether there is indeed a genetic causal relationship between these specific behaviors of smoking, alcohol intake, coffee consumption, and arthritis at the genetic level and the magnitude of the causal relationship remains unclear. Our study clarified the role of these behaviors in arthritis by pooling analyses and deriving a more reliable estimate of genetic causal effects from different studies on arthritis, combined with past research [72]. Our findings may be useful for guiding clinical efforts in identifying and validating new disease prevention and treatment options to improve disease prognosis. Our findings suggest that osteoarthritis-susceptible people and patients should reduce smoking, for RA, and also reduce coffee consumption. We did not find a genetic causal relationship between alcohol intake and arthritis and therefore do not support the idea that alcohol is beneficial for arthritis. Furthermore, our study provides potential research directions for exploring the pathogenesis of arthritis.

### 4.6. Further Studies

In the future, GWAS cohorts with different regional populations, races, and behaviors will be further enriched, and MR studies will be available to determine additional genetic causal relationships between lifestyle habits as risk factors for diseases. Meanwhile, for the identified risk factors such as alcohol intake, smoking, and coffee consumption, exploring their mechanisms of action in the development of arthritis will be the next research direction.

## 5. Conclusions

This study performed a meta-analysis of lifestyle and arthritis and showed positive genetic causality between smoking and coffee consumption with arthritis (OA and RA), while there was insufficient evidence of genetic causality between alcohol intake and arthritis. The results provide a basis for tertiary prevention in clinical practice and a potential direction for further arthritis-related research.

## Data Availability

The study was based on published publications and public databases. The datasets can be found on the IFMRS website (https://msk.hugeamp.org/), the FINNGEN website (https://www.finngen.fi/en), and the IEU OpenGWAS project (gwas.mrcieu.ac.uk).

## Supplementary Materials

The following supporting information can be downloaded at: https://www.mdpi.com/article/10.3390/nu15235009/s1, Table S1. Results of MR analysis of alcoholic drinks per week on five arthritis outcomes. Table S2. Results of MR analysis of alcohol intake frequency on five arthritis outcomes. Table S3. Results of MR analysis of smoking initiation on five arthritis outcomes. Table S4. Results of MR analysis of Coffee intake on five arthritis outcomes. Table S5. Sensitivity analysis of MR results. Table S6. Details of SNPs used for MR analysis of alcoholic drinks per week on HOA. Table S7. Details of SNPs used for MR analysis of alcoholic drinks per week on KOA. Table S8. Details of SNPs used for MR analysis of alcoholic drinks per week on RA (M13). Table S9. Details of SNPs used for MR analysis of alcoholic drinks per week on Seronegative RA. Table S10. Details of SNPs used for MR analysis of alcoholic drinks per week on Seropositive RA. Table S11. Details of SNPs used for MR analysis of alcohol intake frequency on HOA. Table S12. Details of SNPs used for MR analysis of alcohol intake frequency on KOA. Table S13. Details of SNPs used for MR analysis of alcohol intake frequency on RA (M13). Table S14. Details of SNPs used for MR analysis of alcohol intake frequency on Seronegative RA. Table S15. Details of SNPs used for MR analysis of alcohol intake frequency on Seropositive RA. Table S16. Details of SNPs used for MR analysis of smoking initiation on HOA. Table S17. Details of SNPs used for MR analysis of smoking initiation on KOA. Table S18. Details of SNPs used for MR analysis of smoking initiation on RA (M13). Table S19. Details of SNPs used for MR analysis of smoking initiation on Seronegative RA. Table S20. Details of SNPs used for MR analysis of smoking initiation on Seropositive RA. Table S21. Details of SNPs used for MR analysis of coffee intake on HOA. Table S22. Details of SNPs used for MR analysis of coffee intake on KOA. Table S23. Details of SNPs used for MR analysis of coffee intake on RA (M13). Table S24. Details of SNPs used for MR analysis of coffee intake on Seronegative RA. Table S25. Details of SNPs used for MR analysis of coffee intake on Seropositive RA.

## Appendix A

**Table A1:** The characteristics of all 11 studies included in the meta-analysis.

Study	Year	Ethnicity	Cohort	Design of MR Study	Genetic Instrument	Exposure	Outcome	Sample Size	Findings
Bae et al. (a) [17]	2018	European descent	UK Biobank	Two-sample univariable MR analysis	24 SNPs associated with alcohol intake frequency as the IVs	Alcohol consumption	Rheumatoid arthritis	5539 autoantibody-positive individuals with RA and 20,169 controls	Results do not support a causal association between alcohol intake and RA risk
Jiang et al. [18]	2021	European descent	European ancestries	Two-sample univariable MR analysis	99 genome-wide significant variants associated with drink	Alcohol consumption	Rheumatoid arthritis	29,880 RA cases and 73,758 controls	No evidence of a causal relationship was identified for RA
Lee (a) [19]	2018	European descent	arcOGEN	Two-sample univariable MR analysis	4 SNPs associated with smoking behavior were selected as IVs	Smoking behavior	Osteoarthritis	7410 cases and 11,009 controls	MR analysis suggested the inverse association between smoking behavior and osteoarthritis
Ni et al. [20]	2022	European descent	UK Biobank	Two-sample univariable MR analysis and multivariable MR (MVMR).	298 independent SNPs were included as IVs for smoking initiation	Smoking initiation	Osteoarthritis	*N* = 327,918	The findings support an independent deleterious causal effect for smoking upon OA risk
Gill et al. [21]	2021	European descent	UK Biobank	Two-sample univariable MR analysis and multivariable MR (MVMR).	24 SNPs associated with smoking as the IVs	Smoking behavior	Osteoarthritis	60,800 OA cases and 328,251 controls	There was evidence of an unfavorable effect of genetically predicted smoking on OA risk in the main IVW MR analyses
Qian et al. [22]	2020	European descent	European ancestries	Two-sample univariable MR analysis	367 SNPs were used as IVs for smoking initiation	Smoking behavior	Rheumatoid arthritis	14,361 cases and 43,923 controls	Results provide support for a causal association between smoking and increased risk of RA
Zhao et al. [23]	2021	European descent	European ancestries	Two sample. Univariable and multivariable MR methods	124 SNPs were used as IVs for Lifetime smoking	Lifetime smoking	Rheumatoid arthritis	14,361 cases, 43,923 controls	Each S.D. increase in smoking exposure led to a higher risk of RA
Zhang et al. [24]	2021	European descent	UK Biobank	Two-sample univariable MR analysis	11 and 8 independent SNPs with moderate LD were selected as genetic instruments for coffee consumption	Coffee consumption	Osteoarthritis	primary outcome, *N* = 50,508 (10,083 with cases and 40,425 controls) secondary outcomes, knee OA (4462 cases and 17,885 controls) and hip OA (12,625 cases and 50,898 controls), and self-reported OA (12,658 cases and 50,898 controls)	Coffee consumption was associated with an increased risk of overall OA, knee OA, self-reported OA, but not hip OA, using primary genetic instruments
Lee (b) [25]	2018	European descent	arcOGEN	Two-sample univariable MR analysis	4 SNPs from coffee consumption GWASs were selected as IVs	Coffee consumption	Osteoarthritis	7410 cases and 11,009 controls	Coffee consumption is causally associated with an increased risk of osteoarthritis
Pu et al. [26]	2022	European descent	UK Biobank	Two-sample univariable MR analysis	27 SNPs related to coffee intake were selected	Coffee consumption	Rheumatoid arthritis	14,361 cases and 43,923 controls	Study did not support a causal association between coffee intake and RA risk
Bae et al. (b) [27]	2018	European descent	European ancestries	Two-sample univariable MR analysis	4 SNPs were selected as IVs	Coffee consumption	Rheumatoid arthritis	*N* = 41,282	Results do not support causal associations between coffee consumption and the development of RA

Sample size and cohort from outcome study. SNP: single nucleotide polymorphisms; IV: instrumental variables; OA: osteoarthritis; RA: rheumatoid arthritis; LD: Linkage Disequilibrium.

**Table A2:** Quality evaluation of the 11 studies included in the meta-analysis.

Study	Title and Abstract	Background	Objectives	Study Design and Data Sources	Statistical Methods and Main Analysis	Assessment of Assumptions	Software and Pre-Registration	Descriptive Data	Main Results	Sensitivity and Additional Analyses	Key Results	Limitations	Interpretation	Generalizability	Total Score	% Score
Bae et al. (a) [17]	1	1	1	0.5	1	1	1	1	1	0.5	1	1	1	0.5	12.5	89.3
Jiang et al. [18]	1	1	1	1	1	1	1	1	1	1	1	1	0.5	1	13.5	96.4
Lee (a) [19]	1	1	1	1	1	0.5	1	0.5	1	1	1	1	1	0.5	12.5	89.3
Ni et al. [20]	1	1	1	1	1	1	1	1	1	1	1	1	1	1	14	100
Gill et al. [21]	1	1	1	1	1	1	1	1	1	1	1	1	1	1	14	100
Qian et al. [22]	1	1	1	1	1	1	1	1	1	1	1	1	1	1	14	100
Zhao et al. [23]	1	1	1	1	1	1	1	1	1	1	1	1	0	1	13	92.9
Zhang et al. [24]	1	1	1	1	1	1	1	0.5	1	1	1	1	1	0.5	13	92.9
Lee (b) [25]	1	1	1	1	1	0.5	1	0.5	1	1	1	1	0.5	0.5	12	85.7
Pu et al. [26]	1	1	1	1	1	1	1	1	1	1	1	1	1	1	14	100
Bae et al. (b) [27]	1	1	1	1	1	0.5	1	0.5	1	1	1	1	1	0.5	12.5	89.3
