# Supplementary material for: The Causal Association between Alcohol, Smoking, Coffee Consumption, and the Risk of Arthritis: A Meta-Analysis of Mendelian Randomization Studies
Source: Nutrients. 2023 Dec 4;15(23):5009. doi: 10.3390/nu15235009 (PMC10707754; doi:10.3390/nu15235009)
Supplement: Supplementary file 1 [file nutrients-15-05009-s001.zip › nutrients-2723789-supplementary.pdf]

Supplementary Material

Table S1. Results of MR analysis of alcoholic drinks per week on five arthritis outcomes. 2

Table S2. Results of MR analysis of alcohol intake frequency on five arthritis outcomes. 3

Table S3. Results of MR analysis of smoking initiation on five arthritis outcomes. 4

Table S4. Results of MR analysis of Coffee intake on five arthritis outcomes. 5

Table S5. Sensitivity analysis of MR results. 6

Table S6. Details of SNPs used for MR analysis of alcoholic drinks per week on HOA. 7

Table S7. Details of SNPs used for MR analysis of alcoholic drinks per week on KOA. 8

Table S8. Details of SNPs used for MR analysis of alcoholic drinks per week on RA(M13). 9

Table S9. Details of SNPs used for MR analysis of alcoholic drinks per week on Seronegative RA. 10

Table S10. Details of SNPs used for MR analysis of alcoholic drinks per week on Seropositive RA. 11

Table S11. Details of SNPs used for MR analysis of alcohol intake frequency on HOA. 13

Table S12. Details of SNPs used for MR analysis of alcohol intake frequency on KOA. 16

Table S13. Details of SNPs used for MR analysis of alcohol intake frequency on RA(M13). 18

Table S14. Details of SNPs used for MR analysis of alcohol intake frequency on Seronegative RA. 22

Table S15. Details of SNPs used for MR analysis of alcohol intake frequency on Seropositive RA. 25

Table S16. Details of SNPs used for MR analysis of smoking initiation on HOA. 29

Table S17. Details of SNPs used for MR analysis of smoking initiation on KOA. 32

Table S18. Details of SNPs used for MR analysis of smoking initiation on RA(M13). 34

Table S19. Details of SNPs used for MR analysis of smoking initiation on Seronegative RA. 37

Table S20. Details of SNPs used for MR analysis of smoking initiation on Seropositive RA. 41

Table S21. Details of SNPs used for MR analysis of coffee intake on HOA. 44

Table S22. Details of SNPs used for MR analysis of coffee intake on KOA. 45

Table S23. Details of SNPs used for MR analysis of coffee intake on RA(M13). 46

Table S24. Details of SNPs used for MR analysis of coffee intake on Seronegative RA. 48

Table S25. Details of SNPs used for MR analysis of coffee intake on Seropositive RA. 49

Table S1. Results of MR analysis of alcoholic drinks per week on five arthritis outcomes.

outcome	method	nSNP	OR	95%CI (low)	95%CI (up)	pval
HOA	IVW	19	0.757	0.509	1.125	0.168
	MR Egger	19	0.408	0.126	1.325	0.154
	Weighted median	19	0.807	0.516	1.263	0.348
	Weighted mode	19	0.501	0.282	0.888	0.029
KOA	IVW	21	1.256909	0.898	1.759	0.182
	MR Egger	21	1.171677	0.400	3.432	0.776
	Weighted median	21	1.002451	0.708	1.420	0.989
	Weighted mode	21	0.894365	0.588	1.361	0.608
RA(M13)	IVW	33	0.997041	0.564	1.764	0.992
	MR Egger	33	1.099456	0.290	4.167	0.890
	Weighted median	33	1.322095	0.633	2.763	0.458
	Weighted mode	33	1.560731	0.569	4.280	0.393
Seronegative RA	IVW	33	0.910097	0.391	2.116	0.827
	MR Egger	33	0.405303	0.059	2.802	0.367
	Weighted median	33	0.73729	0.234	2.323	0.603
	Weighted mode	33	0.535519	0.108	2.650	0.450
Seropositive RA	IVW	33	0.745118	0.411	1.350	0.332
	MR Egger	33	0.882867	0.219	3.553	0.862
	Weighted median	33	0.97665	0.437	2.184	0.954
	Weighted mode	33	1.222071	0.404	3.696	0.725

HOA: Hip osteoarthritis; KOA: Knee osteoarthritis; RA: Rheumatoid arthritis; OR: Odds ratio; CI: Confidence interval; pval: significance P-value.

Table S2. Results of MR analysis of alcohol intake frequency on five arthritis outcomes.

outcome	method	nSNP	OR	95%CI (low)	95%CI (up)	pval
HOA	IVW	73	1.057	0.930	1.201	0.395
	MR Egger	73	1.544	1.005	2.371	0.051
	Weighted median	73	1.164	0.987	1.372	0.072
	Weighted mode	73	1.514	1.134	2.021	0.006
KOA	IVW	72	1.001	0.893	1.122	0.987
	MR Egger	72	0.994	0.674	1.467	0.977
	Weighted median	72	1.073	0.928	1.241	0.343
	Weighted mode	72	1.062	0.854	1.320	0.593
RA(M13)	IVW	90	1.078	0.870	1.335	0.493
	MR Egger	90	1.167	0.601	2.267	0.650
	Weighted median	90	1.050	0.769	1.432	0.759
	Weighted mode	90	1.471	0.780	2.775	0.237
Seronegative RA	IVW	92	1.009	0.716	1.421	0.959
	MR Egger	92	1.804	0.626	5.201	0.278
	Weighted median	92	1.304	0.768	2.216	0.326
	Weighted mode	92	1.562	0.691	3.530	0.286
Seropositive RA	IVW	92	1.128	0.875	1.455	0.351
	MR Egger	92	1.198	0.541	2.654	0.657
	Weighted median	92	1.276	0.904	1.802	0.165
	Weighted mode	92	1.410	0.769	2.586	0.269

HOA: Hip osteoarthritis; KOA: Knee osteoarthritis; RA: Rheumatoid arthritis; OR: Odds ratio; CI: Confidence interval; pval: significance P-value.

Table S3. Results of MR analysis of smoking initiation on five arthritis outcomes.

outcome	method	nSNP	OR	95%CI (low)	95%CI (up)	pval
HOA	IVW	65	1.066	0.942	1.207	0.311
	MR Egger	65	1.332	0.663	2.677	0.424
	Weighted median	65	1.043	0.900	1.208	0.580
	Weighted mode	65	1.058	0.792	1.414	0.703
KOA	IVW	62	1.003	0.907	1.108	0.961
	MR Egger	62	1.260	0.734	2.162	0.405
	Weighted median	62	1.012	0.887	1.154	0.860
	Weighted mode	62	1.133	0.923	1.392	0.236
RA(M13)	IVW	83	1.393	1.148	1.691	0.001
	MR Egger	83	1.042	0.394	2.757	0.935
	Weighted median	83	1.521	1.154	2.004	0.003
	Weighted mode	83	1.656	0.949	2.892	0.080
Seronegative RA	IVW	83	1.352	0.994	1.839	0.055
	MR Egger	83	0.716	0.153	3.346	0.672
	Weighted median	83	1.378	0.890	2.134	0.151
	Weighted mode	83	1.440	0.548	3.785	0.461
Seropositive RA	IVW	83	1.320	1.070	1.627	0.009
	MR Egger	83	0.623	0.219	1.773	0.378
	Weighted median	83	1.341	0.987	1.822	0.060
	Weighted mode	83	1.333	0.705	2.523	0.379

HOA: Hip osteoarthritis; KOA: Knee osteoarthritis; RA: Rheumatoid arthritis; OR: Odds ratio; CI: Confidence interval; pval: significance P-value.

Table S4. Results of MR analysis of Coffee intake on five arthritis outcomes.

outcome	method	nSNP	OR	95%CI (low)	95%CI (up)	pval
HOA	IVW	25	0.895	0.582	1.374	0.611
	MR Egger	25	0.384	0.104	1.416	0.164
	Weighted median	25	1.017	0.603	1.716	0.949
	Weighted mode	25	1.214	0.341	4.327	0.767
KOA	IVW	26	0.988	0.735	1.328	0.935
	MR Egger	26	0.622	0.247	1.565	0.323
	Weighted median	26	0.848	0.577	1.247	0.402
	Weighted mode	26	0.655	0.306	1.399	0.285
RA(M13)	IVW	38	1.226	0.721	2.084	0.453
	MR Egger	38	0.976	0.332	2.870	0.965
	Weighted median	38	1.002	0.511	1.964	0.996
	Weighted mode	38	1.086	0.512	2.306	0.831
Seronegative RA	IVW	38	2.231	1.037	4.797	0.040
	MR Egger	38	2.798	0.600	13.049	0.199
	Weighted median	38	4.040	1.354	12.056	0.012
	Weighted mode	38	2.960	0.920	9.524	0.077
Seropositive RA	IVW	38	1.180	0.621	2.243	0.613
	MR Egger	38	0.773	0.211	2.836	0.701
	Weighted median	38	1.423	0.631	3.211	0.395
	Weighted mode	38	1.042	0.449	2.419	0.924

HOA: Hip osteoarthritis; KOA: Knee osteoarthritis; RA: Rheumatoid arthritis; OR: Odds ratio; CI: Confidence interval; pval: significance P-value.

Table S5. Sensitivity analysis of MR results.

		Heterogeneity Test			Pleiotropy Test	MR-PRESSO
		Cochran's Q-Test(p-value)		I^2	Egger Intercept	Distortion test
**Exposure**	**Outcome**	**IVW**	**MR-Egger**	**IVW**	**p-value**	**Outliers**
Alcohol(a)	HOA	<0.05	<0.05	0.48	0.29	NA
	KOA	<0.05	<0.05	0.60	0.89	NA
	RA(M13)	0.06	<0.05	0.29	0.87	NA
	Seronegative RA	0.35	0.34	0.07	0.37	NA
	Seropositive RA	0.25	0.21	0.14	0.79	NA
Alcohol(b)	HOA	<0.05	<0.05	0.33	0.07	NA
	KOA	<0.05	<0.05	0.47	0.97	NA
	RA(M13)	0.14	0.12	0.14	0.80	NA
	Seronegative RA	0.30	0.31	0.07	0.26	NA
	Seropositive RA	<0.05	<0.05	0.22	0.88	NA
smoking	HOA	<0.05	<0.05	0.38	0.53	NA
	KOA	<0.05	<0.05	0.42	0.40	NA
	RA(M13)	0.19	0.18	0.12	0.55	NA
	Seronegative RA	0.98	0.98	0.00	0.41	NA
	Seropositive RA	0.46	0.49	0.01	0.16	NA
Coffee	HOA	<0.05	<0.05	0.39	0.19	NA
	KOA	0.17	0.17	0.21	0.31	NA
	RA(M13)	0.06	0.05	0.27	0.64	NA
	Seronegative RA	0.77	0.73	0.00	0.74	NA

	Seropositive RA	<0.05	<0.05	0.35	0.48	NA

Alcohol(a): Alcoholic drinks per week; Alcohol(b): Alcohol intake frequency; HOA: Hip osteoarthritis; KOA: Knee osteoarthritis; RA: Rheumatoid arthritis;

Table S6. Details of SNPs used for MR analysis of alcoholic drinks per week on HOA.

chr	pos	SNP	effect_allele	other_allele	beta	eaf	se	pval	R^2	F
2	27730940	rs1260326	C	T	0.0238	0.595	1.98E-03	3.33E-33	2.73E-04	145.51
2	44271496	rs75120545	T	C	-0.0328	0.022	5.67E-03	7.59E-09	4.63E-05	21.96
2	45141180	rs494904	C	T	0.0151	0.429	1.96E-03	1.41E-14	1.11E-04	59.21
2	63269604	rs6739804	C	T	-0.0130	0.66	2.08E-03	4.72E-10	7.55E-05	40.18
4	39413780	rs28712821	A	G	0.0283	0.594	1.97E-03	1.10E-46	3.87E-04	203.61
4	42117559	rs16854020	A	G	0.0181	0.127	2.91E-03	4.82E-10	7.25E-05	38.69
4	100279889	rs78234152	A	G	0.0277	0.0986	3.07E-03	2.18E-19	1.36E-04	72.61
5	155902003	rs55872084	T	G	0.0127	0.218	2.27E-03	1.98E-08	5.53E-05	29.27
7	103812171	rs2299409	A	G	-0.0106	0.493	1.93E-03	4.80E-08	5.57E-05	29.75
7	153489725	rs6969458	A	G	0.0127	0.459	1.94E-03	5.20E-11	8.02E-05	40.87
9	108755622	rs55932213	G	A	0.0125	0.701	2.22E-03	1.80E-08	6.53E-05	33.67
11	113412443	rs4309187	C	A	0.0148	0.697	2.09E-03	1.37E-12	9.24E-05	49.08
11	113655696	rs17542254	G	A	0.0131	0.251	2.15E-03	8.96E-10	6.49E-05	34.67
12	92081800	rs1387766	A	G	-0.0108	0.622	1.98E-03	4.79E-08	5.51E-05	29.50
14	57281154	rs962961	T	C	-0.0122	0.329	2.05E-03	2.78E-09	6.56E-05	35.02
16	72338507	rs79616692	C	G	0.0188	0.11	3.15E-03	2.38E-09	6.93E-05	36.68
16	73912503	rs11860773	C	T	-0.0150	0.176	2.44E-03	8.35E-10	6.53E-05	33.66
16	85721809	rs13332432	G	C	0.0140	0.296	2.14E-03	5.94E-11	8.17E-05	42.26
17	7733833	rs34121753	G	A	0.0111	0.532	1.95E-03	1.39E-08	6.10E-05	31.62

Table S7. Details of SNPs used for MR analysis of alcoholic drinks per week on KOA.

chr	pos	SNP	effect_allele	other_allele	beta	eaf	se	pval	R^2	F
2	27730940	rs1260326	C	T	0.0238	0.595	1.98E-03	3.33E-33	2.73E-04	145.51
2	44271496	rs75120545	T	C	-0.0328	0.022	5.67E-03	7.59E-09	4.63E-05	21.96
2	45141180	rs494904	C	T	0.0151	0.429	1.96E-03	1.41E-14	1.11E-04	59.21
2	63269604	rs6739804	C	T	-0.0130	0.66	2.08E-03	4.72E-10	7.55E-05	40.18
4	39413780	rs28712821	A	G	0.0283	0.594	1.97E-03	1.10E-46	3.87E-04	203.61
4	42117559	rs16854020	A	G	0.0181	0.127	2.91E-03	4.82E-10	7.25E-05	38.69
4	100279889	rs78234152	A	G	0.0277	0.0986	3.07E-03	2.18E-19	1.36E-04	72.61
5	155902003	rs55872084	T	G	0.0127	0.218	2.27E-03	1.98E-08	5.53E-05	29.27
7	103812171	rs2299409	A	G	-0.0106	0.493	1.93E-03	4.80E-08	5.57E-05	29.75
7	153489725	rs6969458	A	G	0.0127	0.459	1.94E-03	5.20E-11	8.02E-05	40.87
9	108755622	rs55932213	G	A	0.0125	0.701	2.22E-03	1.80E-08	6.53E-05	33.67
11	113412443	rs4309187	C	A	0.0148	0.697	2.09E-03	1.37E-12	9.24E-05	49.08
11	113655696	rs17542254	G	A	0.0131	0.251	2.15E-03	8.96E-10	6.49E-05	34.67
12	92081800	rs1387766	A	G	-0.0108	0.622	1.98E-03	4.79E-08	5.51E-05	29.50
14	57281154	rs962961	T	C	-0.0122	0.329	2.05E-03	2.78E-09	6.56E-05	35.02
16	72338507	rs79616692	C	G	0.0188	0.11	3.15E-03	2.38E-09	6.93E-05	36.68
16	73912503	rs11860773	C	T	-0.0150	0.176	2.44E-03	8.35E-10	6.53E-05	33.66
16	85721809	rs13332432	G	C	0.0140	0.296	2.14E-03	5.94E-11	8.17E-05	42.26
17	7733833	rs34121753	G	A	0.0111	0.532	1.95E-03	1.39E-08	6.10E-05	31.62
17	44189858	rs76640332	A	G	-0.0210	0.204	2.39E-03	1.47E-18	1.43E-04	73.82
19	49211969	rs676388	C	T	0.0151	0.494	1.93E-03	5.49E-15	1.14E-04	60.63

Table S8. Details of SNPs used for MR analysis of alcoholic drinks per week on RA(M13).

chr	pos	SNP	effect_allele	other_allele	beta	eaf	se	pval	R^2	F
1	173848808	rs28680958	A	G	-0.0136	0.23	2.37E-03	9.78E-09	6.54E-05	34.78
2	27730940	rs1260326	C	T	0.0238	0.595	1.98E-03	3.33E-33	2.73E-04	145.51
2	44271496	rs75120545	T	C	-0.0328	0.022	5.67E-03	7.59E-09	4.63E-05	21.96
2	45141180	rs494904	C	T	0.0151	0.429	1.96E-03	1.41E-14	1.11E-04	59.21
2	63269604	rs6739804	C	T	-0.0130	0.66	2.08E-03	4.72E-10	7.55E-05	40.18
3	85403892	rs28732378	G	A	-0.0167	0.729	2.19E-03	2.24E-14	1.11E-04	58.60
3	85766025	rs9835772	T	A	0.0132	0.235	2.24E-03	3.90E-09	6.27E-05	33.26
4	39413780	rs28712821	A	G	0.0283	0.594	1.97E-03	1.10E-46	3.87E-04	203.61
4	42117559	rs16854020	A	G	0.0181	0.127	2.91E-03	4.82E-10	7.25E-05	38.69
4	100239319	rs1229984	C	T	0.1881	0.953	6.18E-03	1.00E-200	3.17E-03	1636.50
4	100279889	rs78234152	A	G	0.0277	0.0986	3.07E-03	2.18E-19	1.36E-04	72.61
4	103188709	rs13107325	T	C	-0.0365	0.0654	3.91E-03	1.23E-20	1.62E-04	85.82
4	143654889	rs331939	A	G	-0.0119	0.339	2.03E-03	4.50E-09	6.35E-05	34.07
5	155902003	rs55872084	T	G	0.0127	0.218	2.27E-03	1.98E-08	5.53E-05	29.27
7	69783020	rs10085696	G	A	-0.0161	0.201	2.49E-03	1.24E-10	8.28E-05	44.28
7	103812171	rs2299409	A	G	-0.0106	0.493	1.93E-03	4.80E-08	5.57E-05	29.75
8	126500031	rs28601761	G	C	0.0113	0.405	1.96E-03	7.60E-09	6.15E-05	32.18
9	108755622	rs55932213	G	A	0.0125	0.701	2.22E-03	1.80E-08	6.53E-05	33.67
11	27694241	rs2049045	C	G	-0.0138	0.189	2.51E-03	3.97E-08	5.81E-05	30.96
11	47428565	rs4752999	T	C	-0.0146	0.321	2.07E-03	2.03E-12	9.25E-05	48.95
11	113412443	rs4309187	C	A	0.0148	0.697	2.09E-03	1.37E-12	9.24E-05	49.08
11	113655696	rs17542254	G	A	0.0131	0.251	2.15E-03	8.96E-10	6.49E-05	34.67
12	92081800	rs1387766	A	G	-0.0108	0.622	1.98E-03	4.79E-08	5.51E-05	29.50
14	57281154	rs962961	T	C	-0.0122	0.329	2.05E-03	2.78E-09	6.56E-05	35.02
14	94844947	rs28929474	T	C	-0.0477	0.0154	7.14E-03	2.39E-11	6.89E-05	36.43

16	28526897	rs153106	C	T	-0.0136	0.409	1.96E-03	3.63E-12	8.96E-05	47.62
16	72338507	rs79616692	C	G	0.0188	0.11	3.15E-03	2.38E-09	6.93E-05	36.68
16	73912503	rs11860773	C	T	-0.0150	0.176	2.44E-03	8.35E-10	6.53E-05	33.66
16	85721809	rs13332432	G	C	0.0140	0.296	2.14E-03	5.94E-11	8.17E-05	42.26
17	7733833	rs34121753	G	A	0.0111	0.532	1.95E-03	1.39E-08	6.10E-05	31.62
17	44189858	rs76640332	A	G	-0.0210	0.204	2.39E-03	1.47E-18	1.43E-04	73.82
19	49211969	rs676388	C	T	0.0151	0.494	1.93E-03	5.49E-15	1.14E-04	60.63
20	25027630	rs6106989	A	G	0.0109	0.628	1.98E-03	3.81E-08	5.55E-05	28.90

Table S9. Details of SNPs used for MR analysis of alcoholic drinks per week on Seronegative RA.

chr	pos	SNP	effect_allele	other_allele	beta	eaf	se	pval	R^2	F
1	173848808	rs28680958	A	G	-0.0136	0.23	2.37E-03	9.78E-09	6.54E-05	34.78
2	27730940	rs1260326	C	T	0.0238	0.595	1.98E-03	3.33E-33	2.73E-04	145.51
2	44271496	rs75120545	T	C	-0.0328	0.022	5.67E-03	7.59E-09	4.63E-05	21.96
2	45141180	rs494904	C	T	0.0151	0.429	1.96E-03	1.41E-14	1.11E-04	59.21
2	63269604	rs6739804	C	T	-0.0130	0.66	2.08E-03	4.72E-10	7.55E-05	40.18
3	85403892	rs28732378	G	A	-0.0167	0.729	2.19E-03	2.24E-14	1.11E-04	58.60
3	85766025	rs9835772	T	A	0.0132	0.235	2.24E-03	3.90E-09	6.27E-05	33.26
4	39413780	rs28712821	A	G	0.0283	0.594	1.97E-03	1.10E-46	3.87E-04	203.61
4	42117559	rs16854020	A	G	0.0181	0.127	2.91E-03	4.82E-10	7.25E-05	38.69
4	100239319	rs1229984	C	T	0.1881	0.953	6.18E-03	1.00E-200	3.17E-03	1636.50
4	100279889	rs78234152	A	G	0.0277	0.0986	3.07E-03	2.18E-19	1.36E-04	72.61
4	103188709	rs13107325	T	C	-0.0365	0.0654	3.91E-03	1.23E-20	1.62E-04	85.82
4	143654889	rs331939	A	G	-0.0119	0.339	2.03E-03	4.50E-09	6.35E-05	34.07
5	155902003	rs55872084	T	G	0.0127	0.218	2.27E-03	1.98E-08	5.53E-05	29.27
7	69783020	rs10085696	G	A	-0.0161	0.201	2.49E-03	1.24E-10	8.28E-05	44.28
7	103812171	rs2299409	A	G	-0.0106	0.493	1.93E-03	4.80E-08	5.57E-05	29.75

8	126500031	rs28601761	G	C	0.0113	0.405	1.96E-03	7.60E-09	6.15E-05	32.18
9	108755622	rs55932213	G	A	0.0125	0.701	2.22E-03	1.80E-08	6.53E-05	33.67
11	27694241	rs2049045	C	G	-0.0138	0.189	2.51E-03	3.97E-08	5.81E-05	30.96
11	47428565	rs4752999	T	C	-0.0146	0.321	2.07E-03	2.03E-12	9.25E-05	48.95
11	113412443	rs4309187	C	A	0.0148	0.697	2.09E-03	1.37E-12	9.24E-05	49.08
11	113655696	rs17542254	G	A	0.0131	0.251	2.15E-03	8.96E-10	6.49E-05	34.67
12	92081800	rs1387766	A	G	-0.0108	0.622	1.98E-03	4.79E-08	5.51E-05	29.50
14	57281154	rs962961	T	C	-0.0122	0.329	2.05E-03	2.78E-09	6.56E-05	35.02
14	94844947	rs28929474	T	C	-0.0477	0.0154	7.14E-03	2.39E-11	6.89E-05	36.43
16	28526897	rs153106	C	T	-0.0136	0.409	1.96E-03	3.63E-12	8.96E-05	47.62
16	72338507	rs79616692	C	G	0.0188	0.11	3.15E-03	2.38E-09	6.93E-05	36.68
16	73912503	rs11860773	C	T	-0.0150	0.176	2.44E-03	8.35E-10	6.53E-05	33.66
16	85721809	rs13332432	G	C	0.0140	0.296	2.14E-03	5.94E-11	8.17E-05	42.26
17	7733833	rs34121753	G	A	0.0111	0.532	1.95E-03	1.39E-08	6.10E-05	31.62
17	44189858	rs76640332	A	G	-0.0210	0.204	2.39E-03	1.47E-18	1.43E-04	73.82
19	49211969	rs676388	C	T	0.0151	0.494	1.93E-03	5.49E-15	1.14E-04	60.63
20	25027630	rs6106989	A	G	0.0109	0.628	1.98E-03	3.81E-08	5.55E-05	28.90

Table S10. Details of SNPs used for MR analysis of alcoholic drinks per week on Seropositive RA.

chr	pos	SNP	effect_allele	other_allele	beta	eaf	se	pval	R^2	F
1	173848808	rs28680958	A	G	-0.0136	0.23	2.37E-03	9.78E-09	6.54E-05	34.78
2	27730940	rs1260326	C	T	0.0238	0.595	1.98E-03	3.33E-33	2.73E-04	145.51
2	44271496	rs75120545	T	C	-0.0328	0.022	5.67E-03	7.59E-09	4.63E-05	21.96
2	45141180	rs494904	C	T	0.0151	0.429	1.96E-03	1.41E-14	1.11E-04	59.21
2	63269604	rs6739804	C	T	-0.0130	0.66	2.08E-03	4.72E-10	7.55E-05	40.18
3	85403892	rs28732378	G	A	-0.0167	0.729	2.19E-03	2.24E-14	1.11E-04	58.60

3	85766025	rs9835772	T	A	0.0132	0.235	2.24E-03	3.90E-09	6.27E-05	33.26
4	39413780	rs28712821	A	G	0.0283	0.594	1.97E-03	1.10E-46	3.87E-04	203.61
4	42117559	rs16854020	A	G	0.0181	0.127	2.91E-03	4.82E-10	7.25E-05	38.69
4	100239319	rs1229984	C	T	0.1881	0.953	6.18E-03	1.00E-200	3.17E-03	1636.50
4	100279889	rs78234152	A	G	0.0277	0.0986	3.07E-03	2.18E-19	1.36E-04	72.61
4	103188709	rs13107325	T	C	-0.0365	0.0654	3.91E-03	1.23E-20	1.62E-04	85.82
4	143654889	rs331939	A	G	-0.0119	0.339	2.03E-03	4.50E-09	6.35E-05	34.07
5	155902003	rs55872084	T	G	0.0127	0.218	2.27E-03	1.98E-08	5.53E-05	29.27
7	69783020	rs10085696	G	A	-0.0161	0.201	2.49E-03	1.24E-10	8.28E-05	44.28
7	103812171	rs2299409	A	G	-0.0106	0.493	1.93E-03	4.80E-08	5.57E-05	29.75
8	126500031	rs28601761	G	C	0.0113	0.405	1.96E-03	7.60E-09	6.15E-05	32.18
9	108755622	rs55932213	G	A	0.0125	0.701	2.22E-03	1.80E-08	6.53E-05	33.67
11	27694241	rs2049045	C	G	-0.0138	0.189	2.51E-03	3.97E-08	5.81E-05	30.96
11	47428565	rs4752999	T	C	-0.0146	0.321	2.07E-03	2.03E-12	9.25E-05	48.95
11	113412443	rs4309187	C	A	0.0148	0.697	2.09E-03	1.37E-12	9.24E-05	49.08
11	113655696	rs17542254	G	A	0.0131	0.251	2.15E-03	8.96E-10	6.49E-05	34.67
12	92081800	rs1387766	A	G	-0.0108	0.622	1.98E-03	4.79E-08	5.51E-05	29.50
14	57281154	rs962961	T	C	-0.0122	0.329	2.05E-03	2.78E-09	6.56E-05	35.02
14	94844947	rs28929474	T	C	-0.0477	0.0154	7.14E-03	2.39E-11	6.89E-05	36.43
16	28526897	rs153106	C	T	-0.0136	0.409	1.96E-03	3.63E-12	8.96E-05	47.62
16	72338507	rs79616692	C	G	0.0188	0.11	3.15E-03	2.38E-09	6.93E-05	36.68
16	73912503	rs11860773	C	T	-0.0150	0.176	2.44E-03	8.35E-10	6.53E-05	33.66
16	85721809	rs13332432	G	C	0.0140	0.296	2.14E-03	5.94E-11	8.17E-05	42.26
17	7733833	rs34121753	G	A	0.0111	0.532	1.95E-03	1.39E-08	6.10E-05	31.62
17	44189858	rs76640332	A	G	-0.0210	0.204	2.39E-03	1.47E-18	1.43E-04	73.82
19	49211969	rs676388	C	T	0.0151	0.494	1.93E-03	5.49E-15	1.14E-04	60.63
20	25027630	rs6106989	A	G	0.0109	0.628	1.98E-03	3.81E-08	5.55E-05	28.90

Table S11. Details of SNPs used for MR analysis of alcohol intake frequency on HOA.

chr	pos	SNP	effect_allele	other_allele	beta	eaf	se	pval	R^2	F
1	940096	rs4503294	T	C	0.0181	0.565	3.07E-03	3.40E-09	1.62E-04	74.85
1	4569436	rs780569	A	T	0.0198	0.709	3.36E-03	4.00E-09	1.62E-04	74.86
1	35363679	rs4417025	A	G	-0.0188	0.361	3.17E-03	2.70E-09	1.64E-04	75.72
1	51218695	rs28787109	A	G	0.0178	0.404	3.08E-03	7.70E-09	1.53E-04	70.65
1	94051350	rs7514579	C	A	0.0197	0.232	3.60E-03	4.60E-08	1.38E-04	63.82
1	216681000	rs2244598	C	T	-0.0184	0.605	3.12E-03	3.80E-09	1.61E-04	74.64
2	2220795	rs72769229	T	A	-0.0231	0.155	4.19E-03	3.40E-08	1.40E-04	64.81
2	23887437	rs1991083	T	C	-0.0224	0.680	3.26E-03	6.30E-12	2.18E-04	100.93
2	27741237	rs780094	C	T	-0.0510	0.615	3.11E-03	1.30E-60	1.23E-03	569.92
2	45139779	rs473098	T	C	-0.0217	0.558	3.04E-03	9.10E-13	2.33E-04	107.83
2	58110969	rs2717063	A	C	-0.0204	0.586	3.08E-03	4.00E-11	2.01E-04	93.12
2	65558588	rs6727281	T	C	-0.0243	0.184	3.92E-03	5.50E-10	1.78E-04	82.15
2	74226102	rs4241258	T	C	0.0251	0.138	4.40E-03	1.30E-08	1.49E-04	68.95
2	97797680	rs13390019	C	T	0.0296	0.134	4.49E-03	4.30E-11	2.04E-04	94.13
2	193989223	rs17662759	C	T	0.0301	0.089	5.46E-03	3.40E-08	1.47E-04	68.17
2	215402926	rs10188314	T	C	-0.0198	0.471	3.04E-03	7.20E-11	1.95E-04	90.22
3	38569463	rs9829192	T	G	0.0169	0.435	3.05E-03	2.80E-08	1.41E-04	65.17
3	49029468	rs76082653	T	C	0.0464	0.054	6.69E-03	3.80E-12	2.21E-04	102.42
3	68408109	rs262240	T	C	-0.0172	0.469	3.03E-03	1.40E-08	1.47E-04	68.18
3	71579022	rs7610856	A	C	-0.0239	0.429	3.07E-03	7.70E-15	2.79E-04	129.04
3	174213976	rs1515591	G	T	0.0182	0.383	3.12E-03	4.90E-09	1.57E-04	72.65
4	39414993	rs11940694	G	A	-0.0437	0.604	3.12E-03	1.00E-44	9.14E-04	422.95
4	55088093	rs28622224	T	C	-0.0186	0.280	3.37E-03	3.20E-08	1.40E-04	64.69

4	100290815	rs62305780	G	C	-0.0485	0.102	5.07E-03	9.90E-22	4.32E-04	199.93
4	135900688	rs13102973	C	T	-0.0194	0.619	3.12E-03	4.90E-10	1.78E-04	82.17
4	184828533	rs62339673	A	C	0.0183	0.627	3.15E-03	6.60E-09	1.57E-04	72.41
5	67824690	rs56194430	T	C	0.0225	0.169	4.07E-03	3.10E-08	1.43E-04	66.08
5	87854395	rs4916723	C	A	0.0239	0.421	3.10E-03	1.10E-14	2.80E-04	129.27
5	144136931	rs461599	C	A	-0.0192	0.462	3.04E-03	2.70E-10	1.83E-04	84.65
5	145615275	rs13178443	T	C	-0.0187	0.276	3.39E-03	3.80E-08	1.39E-04	64.34
5	166830787	rs11750777	A	G	-0.0205	0.209	3.73E-03	3.80E-08	1.39E-04	64.31
6	12903957	rs9349379	G	A	-0.0193	0.405	3.08E-03	3.50E-10	1.80E-04	83.44
6	32074804	rs12153855	C	T	0.0294	0.105	4.93E-03	2.40E-09	1.63E-04	75.33
6	141705482	rs9403297	A	G	0.0188	0.373	3.13E-03	1.80E-09	1.66E-04	76.63
7	39325802	rs9648478	A	G	0.0169	0.510	3.03E-03	2.60E-08	1.42E-04	65.70
7	73042085	rs62466318	T	C	-0.0255	0.203	3.77E-03	1.40E-11	2.10E-04	97.18
7	99872071	rs6943160	C	T	0.0206	0.209	3.73E-03	3.10E-08	1.41E-04	64.97
7	141668403	rs4726481	T	G	0.0218	0.401	3.10E-03	2.30E-12	2.27E-04	105.17
7	153486704	rs2622167	A	G	-0.0191	0.429	3.07E-03	4.60E-10	1.79E-04	82.77
8	30840651	rs2160935	T	C	-0.0187	0.604	3.09E-03	1.40E-09	1.68E-04	77.48
8	87214346	rs34440851	T	C	-0.0227	0.157	4.15E-03	4.60E-08	1.36E-04	63.03
8	141539923	rs2977454	G	C	-0.0259	0.124	4.60E-03	1.70E-08	1.46E-04	67.54
9	16287769	rs74679146	C	T	-0.0321	0.075	5.76E-03	2.50E-08	1.42E-04	65.61
10	133761285	rs34473884	A	G	-0.0204	0.248	3.50E-03	6.20E-09	1.55E-04	71.54
11	58394154	rs550942	T	C	0.0224	0.824	3.99E-03	2.00E-08	1.46E-04	67.34
11	82688356	rs10792669	G	A	0.0174	0.505	3.04E-03	9.90E-09	1.52E-04	70.25
11	121801129	rs1666658	C	T	0.0180	0.392	3.10E-03	6.70E-09	1.54E-04	71.17
11	133780757	rs11223617	A	G	0.0251	0.206	3.75E-03	2.30E-11	2.06E-04	95.29
12	23727301	rs7298932	G	A	-0.0237	0.148	4.31E-03	3.80E-08	1.42E-04	65.57
12	57511734	rs12312693	C	T	-0.0177	0.452	3.05E-03	6.80E-09	1.55E-04	71.61

12	123885974	rs28768122	C	T	0.0207	0.760	3.55E-03	5.60E-09	1.57E-04	72.38
13	49971400	rs7330939	T	C	-0.0213	0.720	3.40E-03	3.70E-10	1.83E-04	84.77
13	68080817	rs1937522	G	A	0.0169	0.528	3.03E-03	2.50E-08	1.42E-04	65.81
14	59072226	rs186347	T	G	0.0179	0.463	3.05E-03	4.00E-09	1.60E-04	74.09
14	73523162	rs2535911	T	C	-0.0188	0.355	3.17E-03	2.70E-09	1.63E-04	75.20
15	34659517	rs117799466	C	G	-0.0197	0.337	3.32E-03	3.10E-09	1.73E-04	79.95
15	76508632	rs80292319	C	T	-0.0394	0.058	6.50E-03	1.40E-09	1.69E-04	77.96
16	6172126	rs34631026	T	C	-0.0169	0.446	3.05E-03	2.90E-08	1.41E-04	65.36
16	19982353	rs8043563	C	G	0.0234	0.737	3.47E-03	1.70E-11	2.12E-04	97.83
16	51205819	rs728538	G	T	0.0229	0.169	4.06E-03	1.80E-08	1.47E-04	67.92
16	72105844	rs72787062	A	G	-0.0282	0.163	4.10E-03	6.40E-12	2.17E-04	100.19
17	7615745	rs9906502	A	G	0.0238	0.177	3.96E-03	1.90E-09	1.65E-04	76.24
17	27588806	rs8614	A	C	0.0248	0.183	3.93E-03	2.70E-10	1.83E-04	84.74
17	29735752	rs9912298	C	A	0.0206	0.240	3.59E-03	9.70E-09	1.54E-04	71.43
17	57780943	rs4968391	T	G	-0.0193	0.675	3.23E-03	2.30E-09	1.63E-04	75.35
18	21080859	rs1893659	A	C	-0.0293	0.460	3.05E-03	7.60E-22	4.27E-04	197.62
18	22639237	rs5022348	T	C	0.0203	0.407	3.57E-03	1.40E-08	1.98E-04	91.66
18	38313195	rs2043677	T	C	0.0261	0.146	4.33E-03	1.60E-09	1.70E-04	78.45
18	53125435	rs2924321	A	G	-0.0195	0.540	3.05E-03	1.60E-10	1.89E-04	87.49
19	49248730	rs838145	A	G	0.0220	0.543	3.06E-03	6.70E-13	2.39E-04	110.63
20	35554361	rs6030200	A	G	-0.0195	0.314	3.27E-03	2.40E-09	1.64E-04	76.00
21	34270051	rs11700855	G	A	-0.0298	0.093	5.23E-03	1.20E-08	1.50E-04	69.56
22	24828853	rs71651683	T	C	-0.0705	0.014	1.28E-02	3.60E-08	1.39E-04	64.27

Table S12. Details of SNPs used for MR analysis of alcohol intake frequency on KOA.

chr	pos	SNP	effect_allele	other_allele	beta	eaf	se	pval	R^2	F

1	940096	rs4503294	T	C	0.0181	0.565	3.07E-03	3.40E-09	1.62E-04	74.85
1	4569436	rs780569	A	T	0.0198	0.709	3.36E-03	4.00E-09	1.62E-04	74.86
1	35363679	rs4417025	A	G	-0.0188	0.361	3.17E-03	2.70E-09	1.64E-04	75.72
1	51218695	rs28787109	A	G	0.0178	0.404	3.08E-03	7.70E-09	1.53E-04	70.65
1	94051350	rs7514579	C	A	0.0197	0.232	3.60E-03	4.60E-08	1.38E-04	63.82
1	216681000	rs2244598	C	T	-0.0184	0.605	3.12E-03	3.80E-09	1.61E-04	74.64
2	2220795	rs72769229	T	A	-0.0231	0.155	4.19E-03	3.40E-08	1.40E-04	64.81
2	23887437	rs1991083	T	C	-0.0224	0.680	3.26E-03	6.30E-12	2.18E-04	100.93
2	27741237	rs780094	C	T	-0.0510	0.615	3.11E-03	1.30E-60	1.23E-03	569.92
2	45139779	rs473098	T	C	-0.0217	0.558	3.04E-03	9.10E-13	2.33E-04	107.83
2	58110969	rs2717063	A	C	-0.0204	0.586	3.08E-03	4.00E-11	2.01E-04	93.12
2	65558588	rs6727281	T	C	-0.0243	0.184	3.92E-03	5.50E-10	1.78E-04	82.15
2	74226102	rs4241258	T	C	0.0251	0.138	4.40E-03	1.30E-08	1.49E-04	68.95
2	97797680	rs13390019	C	T	0.0296	0.134	4.49E-03	4.30E-11	2.04E-04	94.13
2	193989223	rs17662759	C	T	0.0301	0.089	5.46E-03	3.40E-08	1.47E-04	68.17
2	215402926	rs10188314	T	C	-0.0198	0.471	3.04E-03	7.20E-11	1.95E-04	90.22
3	38569463	rs9829192	T	G	0.0169	0.435	3.05E-03	2.80E-08	1.41E-04	65.17
3	49029468	rs76082653	T	C	0.0464	0.054	6.69E-03	3.80E-12	2.21E-04	102.42
3	68408109	rs262240	T	C	-0.0172	0.469	3.03E-03	1.40E-08	1.47E-04	68.18
3	71579022	rs7610856	A	C	-0.0239	0.429	3.07E-03	7.70E-15	2.79E-04	129.04
3	174213976	rs1515591	G	T	0.0182	0.383	3.12E-03	4.90E-09	1.57E-04	72.65
4	39414993	rs11940694	G	A	-0.0437	0.604	3.12E-03	1.00E-44	9.14E-04	422.95
4	55088093	rs28622224	T	C	-0.0186	0.280	3.37E-03	3.20E-08	1.40E-04	64.69
4	100290815	rs62305780	G	C	-0.0485	0.102	5.07E-03	9.90E-22	4.32E-04	199.93
4	135900688	rs13102973	C	T	-0.0194	0.619	3.12E-03	4.90E-10	1.78E-04	82.17
4	184828533	rs62339673	A	C	0.0183	0.627	3.15E-03	6.60E-09	1.57E-04	72.41
5	67824690	rs56194430	T	C	0.0225	0.169	4.07E-03	3.10E-08	1.43E-04	66.08

5	87854395	rs4916723	C	A	0.0239	0.421	3.10E-03	1.10E-14	2.80E-04	129.27
5	144136931	rs461599	C	A	-0.0192	0.462	3.04E-03	2.70E-10	1.83E-04	84.65
5	145615275	rs13178443	T	C	-0.0187	0.276	3.39E-03	3.80E-08	1.39E-04	64.34
5	166830787	rs11750777	A	G	-0.0205	0.209	3.73E-03	3.80E-08	1.39E-04	64.31
6	12903957	rs9349379	G	A	-0.0193	0.405	3.08E-03	3.50E-10	1.80E-04	83.44
6	32074804	rs12153855	C	T	0.0294	0.105	4.93E-03	2.40E-09	1.63E-04	75.33
6	141705482	rs9403297	A	G	0.0188	0.373	3.13E-03	1.80E-09	1.66E-04	76.63
7	39325802	rs9648478	A	G	0.0169	0.510	3.03E-03	2.60E-08	1.42E-04	65.70
7	73042085	rs62466318	T	C	-0.0255	0.203	3.77E-03	1.40E-11	2.10E-04	97.18
7	99872071	rs6943160	C	T	0.0206	0.209	3.73E-03	3.10E-08	1.41E-04	64.97
7	141668403	rs4726481	T	G	0.0218	0.401	3.10E-03	2.30E-12	2.27E-04	105.17
8	30840651	rs2160935	T	C	-0.0187	0.604	3.09E-03	1.40E-09	1.68E-04	77.48
8	87214346	rs34440851	T	C	-0.0227	0.157	4.15E-03	4.60E-08	1.36E-04	63.03
8	141539923	rs2977454	G	C	-0.0259	0.124	4.60E-03	1.70E-08	1.46E-04	67.54
9	16287769	rs74679146	C	T	-0.0321	0.075	5.76E-03	2.50E-08	1.42E-04	65.61
10	133761285	rs34473884	A	G	-0.0204	0.248	3.50E-03	6.20E-09	1.55E-04	71.54
11	58394154	rs550942	T	C	0.0224	0.824	3.99E-03	2.00E-08	1.46E-04	67.34
11	82688356	rs10792669	G	A	0.0174	0.505	3.04E-03	9.90E-09	1.52E-04	70.25
11	121801129	rs1666658	C	T	0.0180	0.392	3.10E-03	6.70E-09	1.54E-04	71.17
11	133780757	rs11223617	A	G	0.0251	0.206	3.75E-03	2.30E-11	2.06E-04	95.29
12	23727301	rs7298932	G	A	-0.0237	0.148	4.31E-03	3.80E-08	1.42E-04	65.57
12	57511734	rs12312693	C	T	-0.0177	0.452	3.05E-03	6.80E-09	1.55E-04	71.61
13	49971400	rs7330939	T	C	-0.0213	0.720	3.40E-03	3.70E-10	1.83E-04	84.77
13	68080817	rs1937522	G	A	0.0169	0.528	3.03E-03	2.50E-08	1.42E-04	65.81
14	59072226	rs186347	T	G	0.0179	0.463	3.05E-03	4.00E-09	1.60E-04	74.09
14	73523162	rs2535911	T	C	-0.0188	0.355	3.17E-03	2.70E-09	1.63E-04	75.20

15	34659517	rs117799466	C	G	-0.0197	0.337	3.32E-03	3.10E-09	1.73E-04	79.95
15	76508632	rs80292319	C	T	-0.0394	0.058	6.50E-03	1.40E-09	1.69E-04	77.96
16	6172126	rs34631026	T	C	-0.0169	0.446	3.05E-03	2.90E-08	1.41E-04	65.36
16	19982353	rs8043563	C	G	0.0234	0.737	3.47E-03	1.70E-11	2.12E-04	97.83
16	51205819	rs728538	G	T	0.0229	0.169	4.06E-03	1.80E-08	1.47E-04	67.92
16	72105844	rs72787062	A	G	-0.0282	0.163	4.10E-03	6.40E-12	2.17E-04	100.19
17	7615745	rs9906502	A	G	0.0238	0.177	3.96E-03	1.90E-09	1.65E-04	76.24
17	27588806	rs8614	A	C	0.0248	0.183	3.93E-03	2.70E-10	1.83E-04	84.74
17	29735752	rs9912298	C	A	0.0206	0.240	3.59E-03	9.70E-09	1.54E-04	71.43
17	43925297	rs17690703	T	C	0.0250	0.263	3.43E-03	2.90E-13	2.43E-04	112.27
17	57780943	rs4968391	T	G	-0.0193	0.675	3.23E-03	2.30E-09	1.63E-04	75.35
18	21080859	rs1893659	A	C	-0.0293	0.460	3.05E-03	7.60E-22	4.27E-04	197.62
18	22639237	rs5022348	T	C	0.0203	0.407	3.57E-03	1.40E-08	1.98E-04	91.66
18	38313195	rs2043677	T	C	0.0261	0.146	4.33E-03	1.60E-09	1.70E-04	78.45
18	53125435	rs2924321	A	G	-0.0195	0.540	3.05E-03	1.60E-10	1.89E-04	87.49
19	49248730	rs838145	A	G	0.0220	0.543	3.06E-03	6.70E-13	2.39E-04	110.63
20	35554361	rs6030200	A	G	-0.0195	0.314	3.27E-03	2.40E-09	1.64E-04	76.00
21	34270051	rs11700855	G	A	-0.0298	0.093	5.23E-03	1.20E-08	1.50E-04	69.56
22	24828853	rs71651683	T	C	-0.0705	0.014	1.28E-02	3.60E-08	1.39E-04	64.27

Table S13. Details of SNPs used for MR analysis of alcohol intake frequency on RA(M13).

chr	pos	SNP	effect_allele	other_allele	beta	eaf	se	pval	R^2	F
1	940096	rs4503294	T	C	0.0181	0.565	3.07E-03	3.40E-09	1.62E-04	74.85
1	4569436	rs780569	A	T	0.0198	0.709	3.36E-03	4.00E-09	1.62E-04	74.86
1	35363679	rs4417025	A	G	-0.0188	0.361	3.17E-03	2.70E-09	1.64E-04	75.72
1	51218695	rs28787109	A	G	0.0178	0.404	3.08E-03	7.70E-09	1.53E-04	70.65
1	94051350	rs7514579	C	A	0.0197	0.232	3.60E-03	4.60E-08	1.38E-04	63.82

1	216681000	rs2244598	C	T	-0.0184	0.605	3.12E-03	3.80E-09	1.61E-04	74.64
2	2220795	rs72769229	T	A	-0.0231	0.155	4.19E-03	3.40E-08	1.40E-04	64.81
2	23887437	rs1991083	T	C	-0.0224	0.680	3.26E-03	6.30E-12	2.18E-04	100.93
2	27741237	rs780094	C	T	-0.0510	0.615	3.11E-03	1.30E-60	1.23E-03	569.92
2	45139779	rs473098	T	C	-0.0217	0.558	3.04E-03	9.10E-13	2.33E-04	107.83
2	58110969	rs2717063	A	C	-0.0204	0.586	3.08E-03	4.00E-11	2.01E-04	93.12
2	65558588	rs6727281	T	C	-0.0243	0.184	3.92E-03	5.50E-10	1.78E-04	82.15
2	74226102	rs4241258	T	C	0.0251	0.138	4.40E-03	1.30E-08	1.49E-04	68.95
2	97797680	rs13390019	C	T	0.0296	0.134	4.49E-03	4.30E-11	2.04E-04	94.13
2	193989223	rs17662759	C	T	0.0301	0.089	5.46E-03	3.40E-08	1.47E-04	68.17
2	215402926	rs10188314	T	C	-0.0198	0.471	3.04E-03	7.20E-11	1.95E-04	90.22
3	38569463	rs9829192	T	G	0.0169	0.435	3.05E-03	2.80E-08	1.41E-04	65.17
3	49029468	rs76082653	T	C	0.0464	0.054	6.69E-03	3.80E-12	2.21E-04	102.42
3	68408109	rs262240	T	C	-0.0172	0.469	3.03E-03	1.40E-08	1.47E-04	68.18
3	71579022	rs7610856	A	C	-0.0239	0.429	3.07E-03	7.70E-15	2.79E-04	129.04
3	85407980	rs9814516	T	G	-0.0251	0.237	3.56E-03	1.60E-12	2.28E-04	105.60
3	131634826	rs1228589	A	G	0.0211	0.246	3.53E-03	2.30E-09	1.65E-04	76.18
3	174213976	rs1515591	G	T	0.0182	0.383	3.12E-03	4.90E-09	1.57E-04	72.65
4	3241845	rs362307	T	C	0.0433	0.075	5.80E-03	8.40E-14	2.59E-04	119.72
4	25408838	rs34811474	A	G	-0.0202	0.231	3.59E-03	1.90E-08	1.45E-04	66.85
4	39414993	rs11940694	G	A	-0.0437	0.604	3.12E-03	1.00E-44	9.14E-04	422.95
4	55088093	rs28622224	T	C	-0.0186	0.280	3.37E-03	3.20E-08	1.40E-04	64.69
4	55521017	rs2159935	A	G	-0.0186	0.490	3.03E-03	8.30E-10	1.72E-04	79.74
4	100239319	rs1229984	C	T	-0.2617	0.973	9.18E-03	1.40E-178	3.63E-03	1683.70
4	100290815	rs62305780	G	C	-0.0485	0.102	5.07E-03	9.90E-22	4.32E-04	199.93
4	103198082	rs13135092	G	A	0.0438	0.083	5.50E-03	1.60E-15	2.94E-04	135.98

4	135900688	rs13102973	C	T	-0.0194	0.619	3.12E-03	4.90E-10	1.78E-04	82.17
4	184828533	rs62339673	A	C	0.0183	0.627	3.15E-03	6.60E-09	1.57E-04	72.41
5	67824690	rs56194430	T	C	0.0225	0.169	4.07E-03	3.10E-08	1.43E-04	66.08
5	87854395	rs4916723	C	A	0.0239	0.421	3.10E-03	1.10E-14	2.80E-04	129.27
5	144136931	rs461599	C	A	-0.0192	0.462	3.04E-03	2.70E-10	1.83E-04	84.65
5	145615275	rs13178443	T	C	-0.0187	0.276	3.39E-03	3.80E-08	1.39E-04	64.34
5	166830787	rs11750777	A	G	-0.0205	0.209	3.73E-03	3.80E-08	1.39E-04	64.31
6	12903957	rs9349379	G	A	-0.0193	0.405	3.08E-03	3.50E-10	1.80E-04	83.44
6	98344031	rs9372625	A	G	-0.0256	0.382	3.12E-03	2.90E-16	3.08E-04	142.59
6	141705482	rs9403297	A	G	0.0188	0.373	3.13E-03	1.80E-09	1.66E-04	76.63
7	1961882	rs73050128	A	C	-0.0260	0.164	4.09E-03	2.10E-10	1.86E-04	85.95
7	39325802	rs9648478	A	G	0.0169	0.510	3.03E-03	2.60E-08	1.42E-04	65.70
7	73042085	rs62466318	T	C	-0.0255	0.203	3.77E-03	1.40E-11	2.10E-04	97.18
7	141668403	rs4726481	T	G	0.0218	0.401	3.10E-03	2.30E-12	2.27E-04	105.17
7	153486704	rs2622167	A	G	-0.0191	0.429	3.07E-03	4.60E-10	1.79E-04	82.77
8	30840651	rs2160935	T	C	-0.0187	0.604	3.09E-03	1.40E-09	1.68E-04	77.48
8	87214346	rs34440851	T	C	-0.0227	0.157	4.15E-03	4.60E-08	1.36E-04	63.03
8	141539923	rs2977454	G	C	-0.0259	0.124	4.60E-03	1.70E-08	1.46E-04	67.54
8	142615222	rs11787216	T	C	0.0244	0.369	3.20E-03	2.40E-14	2.78E-04	128.41
9	16287769	rs74679146	C	T	-0.0321	0.075	5.76E-03	2.50E-08	1.42E-04	65.61
10	99715744	rs489062	A	G	0.0166	0.437	3.05E-03	4.90E-08	1.36E-04	63.09
10	102626510	rs61873510	T	G	0.0204	0.328	3.30E-03	6.90E-10	1.83E-04	84.60
10	133761285	rs34473884	A	G	-0.0204	0.248	3.50E-03	6.20E-09	1.55E-04	71.54
10	133986135	rs4242715	A	G	-0.0187	0.681	3.25E-03	9.30E-09	1.51E-04	69.96
11	47867059	rs11039429	T	C	-0.0236	0.455	3.04E-03	8.70E-15	2.75E-04	127.29
11	58394154	rs550942	T	C	0.0224	0.824	3.99E-03	2.00E-08	1.46E-04	67.34
11	82688356	rs10792669	G	A	0.0174	0.505	3.04E-03	9.90E-09	1.52E-04	70.25

11	121801129	rs1666658	C	T	0.0180	0.392	3.10E-03	6.70E-09	1.54E-04	71.17
11	133780757	rs11223617	A	G	0.0251	0.206	3.75E-03	2.30E-11	2.06E-04	95.29
12	23727301	rs7298932	G	A	-0.0237	0.148	4.31E-03	3.80E-08	1.42E-04	65.57
12	54623132	rs58905411	A	G	-0.0266	0.410	3.08E-03	5.10E-18	3.43E-04	158.74
12	56449435	rs7302200	A	G	-0.0184	0.340	3.20E-03	8.40E-09	1.52E-04	70.43
12	57511734	rs12312693	C	T	-0.0177	0.452	3.05E-03	6.80E-09	1.55E-04	71.61
12	123885974	rs28768122	C	T	0.0207	0.760	3.55E-03	5.60E-09	1.57E-04	72.38
13	49971400	rs7330939	T	C	-0.0213	0.720	3.40E-03	3.70E-10	1.83E-04	84.77
13	68080817	rs1937522	G	A	0.0169	0.528	3.03E-03	2.50E-08	1.42E-04	65.81
14	59072226	rs186347	T	G	0.0179	0.463	3.05E-03	4.00E-09	1.60E-04	74.09
14	73523162	rs2535911	T	C	-0.0188	0.355	3.17E-03	2.70E-09	1.63E-04	75.20
15	76508632	rs80292319	C	T	-0.0394	0.058	6.50E-03	1.40E-09	1.69E-04	77.96
16	6172126	rs34631026	T	C	-0.0169	0.446	3.05E-03	2.90E-08	1.41E-04	65.36
16	19982353	rs8043563	C	G	0.0234	0.737	3.47E-03	1.70E-11	2.12E-04	97.83
16	28632021	rs2411453	G	T	-0.0351	0.597	3.09E-03	7.30E-30	5.92E-04	273.85
16	30057148	rs35105141	T	C	0.0263	0.402	3.09E-03	1.40E-17	3.34E-04	154.28
16	51205819	rs728538	G	T	0.0229	0.169	4.06E-03	1.80E-08	1.47E-04	67.92
16	53800954	rs1421085	C	T	0.0199	0.403	3.08E-03	1.00E-10	1.91E-04	88.50
16	72105844	rs72787062	A	G	-0.0282	0.163	4.10E-03	6.40E-12	2.17E-04	100.19
17	7615745	rs9906502	A	G	0.0238	0.177	3.96E-03	1.90E-09	1.65E-04	76.24
17	27588806	rs8614	A	C	0.0248	0.183	3.93E-03	2.70E-10	1.83E-04	84.74
17	29735752	rs9912298	C	A	0.0206	0.240	3.59E-03	9.70E-09	1.54E-04	71.43
17	40721042	rs650558	T	C	0.0207	0.248	3.51E-03	3.40E-09	1.60E-04	74.15
17	43925297	rs17690703	T	C	0.0250	0.263	3.43E-03	2.90E-13	2.43E-04	112.27
17	57780943	rs4968391	T	G	-0.0193	0.675	3.23E-03	2.30E-09	1.63E-04	75.35
18	22639237	rs5022348	T	C	0.0203	0.407	3.57E-03	1.40E-08	1.98E-04	91.66

18	38269812	rs9958320	C	T	0.0249	0.153	4.27E-03	5.90E-09	1.60E-04	74.10
18	53125435	rs2924321	A	G	-0.0195	0.540	3.05E-03	1.60E-10	1.89E-04	87.49
19	49248730	rs838145	A	G	0.0220	0.543	3.06E-03	6.70E-13	2.39E-04	110.63
20	35554361	rs6030200	A	G	-0.0195	0.314	3.27E-03	2.40E-09	1.64E-04	76.00
21	34270051	rs11700855	G	A	-0.0298	0.093	5.23E-03	1.20E-08	1.50E-04	69.56
22	24828853	rs71651683	T	C	-0.0705	0.014	1.28E-02	3.60E-08	1.39E-04	64.27

Table S14. Details of SNPs used for MR analysis of alcohol intake frequency on Seronegative RA.

chr	pos	SNP	effect_allele	other_allele	beta	eaf	se	pval	R^2	F
1	940096	rs4503294	T	C	0.0181	0.565	3.07E-03	3.40E-09	1.62E-04	74.85
1	4569436	rs780569	A	T	0.0198	0.709	3.36E-03	4.00E-09	1.62E-04	74.86
1	35363679	rs4417025	A	G	-0.0188	0.361	3.17E-03	2.70E-09	1.64E-04	75.72
1	51218695	rs28787109	A	G	0.0178	0.404	3.08E-03	7.70E-09	1.53E-04	70.65
1	94051350	rs7514579	C	A	0.0197	0.232	3.60E-03	4.60E-08	1.38E-04	63.82
1	216681000	rs2244598	C	T	-0.0184	0.605	3.12E-03	3.80E-09	1.61E-04	74.64
2	2220795	rs72769229	T	A	-0.0231	0.155	4.19E-03	3.40E-08	1.40E-04	64.81
2	23887437	rs1991083	T	C	-0.0224	0.680	3.26E-03	6.30E-12	2.18E-04	100.93
2	27741237	rs780094	C	T	-0.0510	0.615	3.11E-03	1.30E-60	1.23E-03	569.92
2	45139779	rs473098	T	C	-0.0217	0.558	3.04E-03	9.10E-13	2.33E-04	107.83
2	58110969	rs2717063	A	C	-0.0204	0.586	3.08E-03	4.00E-11	2.01E-04	93.12
2	65558588	rs6727281	T	C	-0.0243	0.184	3.92E-03	5.50E-10	1.78E-04	82.15
2	74226102	rs4241258	T	C	0.0251	0.138	4.40E-03	1.30E-08	1.49E-04	68.95
2	97797680	rs13390019	C	T	0.0296	0.134	4.49E-03	4.30E-11	2.04E-04	94.13
2	193989223	rs17662759	C	T	0.0301	0.089	5.46E-03	3.40E-08	1.47E-04	68.17
2	215402926	rs10188314	T	C	-0.0198	0.471	3.04E-03	7.20E-11	1.95E-04	90.22
3	38569463	rs9829192	T	G	0.0169	0.435	3.05E-03	2.80E-08	1.41E-04	65.17
3	49029468	rs76082653	T	C	0.0464	0.054	6.69E-03	3.80E-12	2.21E-04	102.42

3	68408109	rs262240	T	C	-0.0172	0.469	3.03E-03	1.40E-08	1.47E-04	68.18
3	71579022	rs7610856	A	C	-0.0239	0.429	3.07E-03	7.70E-15	2.79E-04	129.04
3	85407980	rs9814516	T	G	-0.0251	0.237	3.56E-03	1.60E-12	2.28E-04	105.60
3	131634826	rs1228589	A	G	0.0211	0.246	3.53E-03	2.30E-09	1.65E-04	76.18
3	174213976	rs1515591	G	T	0.0182	0.383	3.12E-03	4.90E-09	1.57E-04	72.65
4	3241845	rs362307	T	C	0.0433	0.075	5.80E-03	8.40E-14	2.59E-04	119.72
4	25408838	rs34811474	A	G	-0.0202	0.231	3.59E-03	1.90E-08	1.45E-04	66.85
4	39414993	rs11940694	G	A	-0.0437	0.604	3.12E-03	1.00E-44	9.14E-04	422.95
4	55088093	rs28622224	T	C	-0.0186	0.280	3.37E-03	3.20E-08	1.40E-04	64.69
4	55521017	rs2159935	A	G	-0.0186	0.490	3.03E-03	8.30E-10	1.72E-04	79.74
4	100239319	rs1229984	C	T	-0.2617	0.973	9.18E-03	1.40E-178	3.63E-03	1683.70
4	100290815	rs62305780	G	C	-0.0485	0.102	5.07E-03	9.90E-22	4.32E-04	199.93
4	103198082	rs13135092	G	A	0.0438	0.083	5.50E-03	1.60E-15	2.94E-04	135.98
4	135900688	rs13102973	C	T	-0.0194	0.619	3.12E-03	4.90E-10	1.78E-04	82.17
4	184828533	rs62339673	A	C	0.0183	0.627	3.15E-03	6.60E-09	1.57E-04	72.41
5	67824690	rs56194430	T	C	0.0225	0.169	4.07E-03	3.10E-08	1.43E-04	66.08
5	87854395	rs4916723	C	A	0.0239	0.421	3.10E-03	1.10E-14	2.80E-04	129.27
5	144136931	rs461599	C	A	-0.0192	0.462	3.04E-03	2.70E-10	1.83E-04	84.65
5	145615275	rs13178443	T	C	-0.0187	0.276	3.39E-03	3.80E-08	1.39E-04	64.34
5	166830787	rs11750777	A	G	-0.0205	0.209	3.73E-03	3.80E-08	1.39E-04	64.31
6	12903957	rs9349379	G	A	-0.0193	0.405	3.08E-03	3.50E-10	1.80E-04	83.44
6	32074804	rs12153855	C	T	0.0294	0.105	4.93E-03	2.40E-09	1.63E-04	75.33
6	98344031	rs9372625	A	G	-0.0256	0.382	3.12E-03	2.90E-16	3.08E-04	142.59
6	141705482	rs9403297	A	G	0.0188	0.373	3.13E-03	1.80E-09	1.66E-04	76.63
7	1961882	rs73050128	A	C	-0.0260	0.164	4.09E-03	2.10E-10	1.86E-04	85.95
7	39325802	rs9648478	A	G	0.0169	0.510	3.03E-03	2.60E-08	1.42E-04	65.70

7	73042085	rs62466318	T	C	-0.0255	0.203	3.77E-03	1.40E-11	2.10E-04	97.18
7	141668403	rs4726481	T	G	0.0218	0.401	3.10E-03	2.30E-12	2.27E-04	105.17
7	153486704	rs2622167	A	G	-0.0191	0.429	3.07E-03	4.60E-10	1.79E-04	82.77
8	30840651	rs2160935	T	C	-0.0187	0.604	3.09E-03	1.40E-09	1.68E-04	77.48
8	87214346	rs34440851	T	C	-0.0227	0.157	4.15E-03	4.60E-08	1.36E-04	63.03
8	141539923	rs2977454	G	C	-0.0259	0.124	4.60E-03	1.70E-08	1.46E-04	67.54
8	142615222	rs11787216	T	C	0.0244	0.369	3.20E-03	2.40E-14	2.78E-04	128.41
9	16287769	rs74679146	C	T	-0.0321	0.075	5.76E-03	2.50E-08	1.42E-04	65.61
10	99715744	rs489062	A	G	0.0166	0.437	3.05E-03	4.90E-08	1.36E-04	63.09
10	102626510	rs61873510	T	G	0.0204	0.328	3.30E-03	6.90E-10	1.83E-04	84.60
10	133761285	rs34473884	A	G	-0.0204	0.248	3.50E-03	6.20E-09	1.55E-04	71.54
10	133986135	rs4242715	A	G	-0.0187	0.681	3.25E-03	9.30E-09	1.51E-04	69.96
11	47867059	rs11039429	T	C	-0.0236	0.455	3.04E-03	8.70E-15	2.75E-04	127.29
11	58394154	rs550942	T	C	0.0224	0.824	3.99E-03	2.00E-08	1.46E-04	67.34
11	82688356	rs10792669	G	A	0.0174	0.505	3.04E-03	9.90E-09	1.52E-04	70.25
11	121801129	rs1666658	C	T	0.0180	0.392	3.10E-03	6.70E-09	1.54E-04	71.17
11	133780757	rs11223617	A	G	0.0251	0.206	3.75E-03	2.30E-11	2.06E-04	95.29
12	23727301	rs7298932	G	A	-0.0237	0.148	4.31E-03	3.80E-08	1.42E-04	65.57
12	54623132	rs58905411	A	G	-0.0266	0.410	3.08E-03	5.10E-18	3.43E-04	158.74
12	56449435	rs7302200	A	G	-0.0184	0.340	3.20E-03	8.40E-09	1.52E-04	70.43
12	57511734	rs12312693	C	T	-0.0177	0.452	3.05E-03	6.80E-09	1.55E-04	71.61
12	123885974	rs28768122	C	T	0.0207	0.760	3.55E-03	5.60E-09	1.57E-04	72.38
13	49971400	rs7330939	T	C	-0.0213	0.720	3.40E-03	3.70E-10	1.83E-04	84.77
13	68080817	rs1937522	G	A	0.0169	0.528	3.03E-03	2.50E-08	1.42E-04	65.81
14	59072226	rs186347	T	G	0.0179	0.463	3.05E-03	4.00E-09	1.60E-04	74.09
14	73523162	rs2535911	T	C	-0.0188	0.355	3.17E-03	2.70E-09	1.63E-04	75.20
15	76508632	rs80292319	C	T	-0.0394	0.058	6.50E-03	1.40E-09	1.69E-04	77.96

16	6172126	rs34631026	T	C	-0.0169	0.446	3.05E-03	2.90E-08	1.41E-04	65.36
16	19982353	rs8043563	C	G	0.0234	0.737	3.47E-03	1.70E-11	2.12E-04	97.83
16	28632021	rs2411453	G	T	-0.0351	0.597	3.09E-03	7.30E-30	5.92E-04	273.85
16	30057148	rs35105141	T	C	0.0263	0.402	3.09E-03	1.40E-17	3.34E-04	154.28
16	51205819	rs728538	G	T	0.0229	0.169	4.06E-03	1.80E-08	1.47E-04	67.92
16	53800954	rs1421085	C	T	0.0199	0.403	3.08E-03	1.00E-10	1.91E-04	88.50
16	72105844	rs72787062	A	G	-0.0282	0.163	4.10E-03	6.40E-12	2.17E-04	100.19
17	7615745	rs9906502	A	G	0.0238	0.177	3.96E-03	1.90E-09	1.65E-04	76.24
17	27588806	rs8614	A	C	0.0248	0.183	3.93E-03	2.70E-10	1.83E-04	84.74
17	29735752	rs9912298	C	A	0.0206	0.240	3.59E-03	9.70E-09	1.54E-04	71.43
17	40721042	rs650558	T	C	0.0207	0.248	3.51E-03	3.40E-09	1.60E-04	74.15
17	43925297	rs17690703	T	C	0.0250	0.263	3.43E-03	2.90E-13	2.43E-04	112.27
17	57780943	rs4968391	T	G	-0.0193	0.675	3.23E-03	2.30E-09	1.63E-04	75.35
18	21080859	rs1893659	A	C	-0.0293	0.460	3.05E-03	7.60E-22	4.27E-04	197.62
18	22639237	rs5022348	T	C	0.0203	0.407	3.57E-03	1.40E-08	1.98E-04	91.66
18	38269812	rs9958320	C	T	0.0249	0.153	4.27E-03	5.90E-09	1.60E-04	74.10
18	53125435	rs2924321	A	G	-0.0195	0.540	3.05E-03	1.60E-10	1.89E-04	87.49
19	49248730	rs838145	A	G	0.0220	0.543	3.06E-03	6.70E-13	2.39E-04	110.63
20	35554361	rs6030200	A	G	-0.0195	0.314	3.27E-03	2.40E-09	1.64E-04	76.00
21	34270051	rs11700855	G	A	-0.0298	0.093	5.23E-03	1.20E-08	1.50E-04	69.56
22	24828853	rs71651683	T	C	-0.0705	0.014	1.28E-02	3.60E-08	1.39E-04	64.27

Table S15. Details of SNPs used for MR analysis of alcohol intake frequency on Seropositive RA.

chr	pos	SNP	effect_allele	other_allele	beta	eaf	se	pval	R^2	F
1	940096	rs4503294	T	C	0.0181	0.565	3.07E-03	3.40E-09	1.62E-04	74.85
1	4569436	rs780569	A	T	0.0198	0.709	3.36E-03	4.00E-09	1.62E-04	74.86

1	35363679	rs4417025	A	G	-0.0188	0.361	3.17E-03	2.70E-09	1.64E-04	75.72
1	51218695	rs28787109	A	G	0.0178	0.404	3.08E-03	7.70E-09	1.53E-04	70.65
1	94051350	rs7514579	C	A	0.0197	0.232	3.60E-03	4.60E-08	1.38E-04	63.82
1	216681000	rs2244598	C	T	-0.0184	0.605	3.12E-03	3.80E-09	1.61E-04	74.64
2	2220795	rs72769229	T	A	-0.0231	0.155	4.19E-03	3.40E-08	1.40E-04	64.81
2	23887437	rs1991083	T	C	-0.0224	0.680	3.26E-03	6.30E-12	2.18E-04	100.93
2	27741237	rs780094	C	T	-0.0510	0.615	3.11E-03	1.30E-60	1.23E-03	569.92
2	45139779	rs473098	T	C	-0.0217	0.558	3.04E-03	9.10E-13	2.33E-04	107.83
2	58110969	rs2717063	A	C	-0.0204	0.586	3.08E-03	4.00E-11	2.01E-04	93.12
2	65558588	rs6727281	T	C	-0.0243	0.184	3.92E-03	5.50E-10	1.78E-04	82.15
2	74226102	rs4241258	T	C	0.0251	0.138	4.40E-03	1.30E-08	1.49E-04	68.95
2	97797680	rs13390019	C	T	0.0296	0.134	4.49E-03	4.30E-11	2.04E-04	94.13
2	193989223	rs17662759	C	T	0.0301	0.089	5.46E-03	3.40E-08	1.47E-04	68.17
2	215402926	rs10188314	T	C	-0.0198	0.471	3.04E-03	7.20E-11	1.95E-04	90.22
3	38569463	rs9829192	T	G	0.0169	0.435	3.05E-03	2.80E-08	1.41E-04	65.17
3	49029468	rs76082653	T	C	0.0464	0.054	6.69E-03	3.80E-12	2.21E-04	102.42
3	68408109	rs262240	T	C	-0.0172	0.469	3.03E-03	1.40E-08	1.47E-04	68.18
3	71579022	rs7610856	A	C	-0.0239	0.429	3.07E-03	7.70E-15	2.79E-04	129.04
3	85407980	rs9814516	T	G	-0.0251	0.237	3.56E-03	1.60E-12	2.28E-04	105.60
3	131634826	rs1228589	A	G	0.0211	0.246	3.53E-03	2.30E-09	1.65E-04	76.18
3	174213976	rs1515591	G	T	0.0182	0.383	3.12E-03	4.90E-09	1.57E-04	72.65
4	3241845	rs362307	T	C	0.0433	0.075	5.80E-03	8.40E-14	2.59E-04	119.72
4	25408838	rs34811474	A	G	-0.0202	0.231	3.59E-03	1.90E-08	1.45E-04	66.85
4	39414993	rs11940694	G	A	-0.0437	0.604	3.12E-03	1.00E-44	9.14E-04	422.95
4	55088093	rs28622224	T	C	-0.0186	0.280	3.37E-03	3.20E-08	1.40E-04	64.69
4	55521017	rs2159935	A	G	-0.0186	0.490	3.03E-03	8.30E-10	1.72E-04	79.74
4	100239319	rs1229984	C	T	-0.2617	0.973	9.18E-03	1.40E-178	3.63E-03	1683.70

4	100290815	rs62305780	G	C	-0.0485	0.102	5.07E-03	9.90E-22	4.32E-04	199.93
4	103198082	rs13135092	G	A	0.0438	0.083	5.50E-03	1.60E-15	2.94E-04	135.98
4	135900688	rs13102973	C	T	-0.0194	0.619	3.12E-03	4.90E-10	1.78E-04	82.17
4	184828533	rs62339673	A	C	0.0183	0.627	3.15E-03	6.60E-09	1.57E-04	72.41
5	67824690	rs56194430	T	C	0.0225	0.169	4.07E-03	3.10E-08	1.43E-04	66.08
5	87854395	rs4916723	C	A	0.0239	0.421	3.10E-03	1.10E-14	2.80E-04	129.27
5	144136931	rs461599	C	A	-0.0192	0.462	3.04E-03	2.70E-10	1.83E-04	84.65
5	145615275	rs13178443	T	C	-0.0187	0.276	3.39E-03	3.80E-08	1.39E-04	64.34
5	166830787	rs11750777	A	G	-0.0205	0.209	3.73E-03	3.80E-08	1.39E-04	64.31
6	12903957	rs9349379	G	A	-0.0193	0.405	3.08E-03	3.50E-10	1.80E-04	83.44
6	32074804	rs12153855	C	T	0.0294	0.105	4.93E-03	2.40E-09	1.63E-04	75.33
6	98344031	rs9372625	A	G	-0.0256	0.382	3.12E-03	2.90E-16	3.08E-04	142.59
6	141705482	rs9403297	A	G	0.0188	0.373	3.13E-03	1.80E-09	1.66E-04	76.63
7	1961882	rs73050128	A	C	-0.0260	0.164	4.09E-03	2.10E-10	1.86E-04	85.95
7	39325802	rs9648478	A	G	0.0169	0.510	3.03E-03	2.60E-08	1.42E-04	65.70
7	73042085	rs62466318	T	C	-0.0255	0.203	3.77E-03	1.40E-11	2.10E-04	97.18
7	141668403	rs4726481	T	G	0.0218	0.401	3.10E-03	2.30E-12	2.27E-04	105.17
7	153486704	rs2622167	A	G	-0.0191	0.429	3.07E-03	4.60E-10	1.79E-04	82.77
8	30840651	rs2160935	T	C	-0.0187	0.604	3.09E-03	1.40E-09	1.68E-04	77.48
8	87214346	rs34440851	T	C	-0.0227	0.157	4.15E-03	4.60E-08	1.36E-04	63.03
8	141539923	rs2977454	G	C	-0.0259	0.124	4.60E-03	1.70E-08	1.46E-04	67.54
8	142615222	rs11787216	T	C	0.0244	0.369	3.20E-03	2.40E-14	2.78E-04	128.41
9	16287769	rs74679146	C	T	-0.0321	0.075	5.76E-03	2.50E-08	1.42E-04	65.61
10	99715744	rs489062	A	G	0.0166	0.437	3.05E-03	4.90E-08	1.36E-04	63.09
10	102626510	rs61873510	T	G	0.0204	0.328	3.30E-03	6.90E-10	1.83E-04	84.60
10	133761285	rs34473884	A	G	-0.0204	0.248	3.50E-03	6.20E-09	1.55E-04	71.54

10	133986135	rs4242715	A	G	-0.0187	0.681	3.25E-03	9.30E-09	1.51E-04	69.96
11	47867059	rs11039429	T	C	-0.0236	0.455	3.04E-03	8.70E-15	2.75E-04	127.29
11	58394154	rs550942	T	C	0.0224	0.824	3.99E-03	2.00E-08	1.46E-04	67.34
11	82688356	rs10792669	G	A	0.0174	0.505	3.04E-03	9.90E-09	1.52E-04	70.25
11	121801129	rs1666658	C	T	0.0180	0.392	3.10E-03	6.70E-09	1.54E-04	71.17
11	133780757	rs11223617	A	G	0.0251	0.206	3.75E-03	2.30E-11	2.06E-04	95.29
12	23727301	rs7298932	G	A	-0.0237	0.148	4.31E-03	3.80E-08	1.42E-04	65.57
12	54623132	rs58905411	A	G	-0.0266	0.410	3.08E-03	5.10E-18	3.43E-04	158.74
12	56449435	rs7302200	A	G	-0.0184	0.340	3.20E-03	8.40E-09	1.52E-04	70.43
12	57511734	rs12312693	C	T	-0.0177	0.452	3.05E-03	6.80E-09	1.55E-04	71.61
12	123885974	rs28768122	C	T	0.0207	0.760	3.55E-03	5.60E-09	1.57E-04	72.38
13	49971400	rs7330939	T	C	-0.0213	0.720	3.40E-03	3.70E-10	1.83E-04	84.77
13	68080817	rs1937522	G	A	0.0169	0.528	3.03E-03	2.50E-08	1.42E-04	65.81
14	59072226	rs186347	T	G	0.0179	0.463	3.05E-03	4.00E-09	1.60E-04	74.09
14	73523162	rs2535911	T	C	-0.0188	0.355	3.17E-03	2.70E-09	1.63E-04	75.20
15	76508632	rs80292319	C	T	-0.0394	0.058	6.50E-03	1.40E-09	1.69E-04	77.96
16	6172126	rs34631026	T	C	-0.0169	0.446	3.05E-03	2.90E-08	1.41E-04	65.36
16	19982353	rs8043563	C	G	0.0234	0.737	3.47E-03	1.70E-11	2.12E-04	97.83
16	28632021	rs2411453	G	T	-0.0351	0.597	3.09E-03	7.30E-30	5.92E-04	273.85
16	30057148	rs35105141	T	C	0.0263	0.402	3.09E-03	1.40E-17	3.34E-04	154.28
16	51205819	rs728538	G	T	0.0229	0.169	4.06E-03	1.80E-08	1.47E-04	67.92
16	53800954	rs1421085	C	T	0.0199	0.403	3.08E-03	1.00E-10	1.91E-04	88.50
16	72105844	rs72787062	A	G	-0.0282	0.163	4.10E-03	6.40E-12	2.17E-04	100.19
17	7615745	rs9906502	A	G	0.0238	0.177	3.96E-03	1.90E-09	1.65E-04	76.24
17	27588806	rs8614	A	C	0.0248	0.183	3.93E-03	2.70E-10	1.83E-04	84.74
17	29735752	rs9912298	C	A	0.0206	0.240	3.59E-03	9.70E-09	1.54E-04	71.43
17	40721042	rs650558	T	C	0.0207	0.248	3.51E-03	3.40E-09	1.60E-04	74.15

17	43925297	rs17690703	T	C	0.0250	0.263	3.43E-03	2.90E-13	2.43E-04	112.27
17	57780943	rs4968391	T	G	-0.0193	0.675	3.23E-03	2.30E-09	1.63E-04	75.35
18	21080859	rs1893659	A	C	-0.0293	0.460	3.05E-03	7.60E-22	4.27E-04	197.62
18	22639237	rs5022348	T	C	0.0203	0.407	3.57E-03	1.40E-08	1.98E-04	91.66
18	38269812	rs9958320	C	T	0.0249	0.153	4.27E-03	5.90E-09	1.60E-04	74.10
18	53125435	rs2924321	A	G	-0.0195	0.540	3.05E-03	1.60E-10	1.89E-04	87.49
19	49248730	rs838145	A	G	0.0220	0.543	3.06E-03	6.70E-13	2.39E-04	110.63
20	35554361	rs6030200	A	G	-0.0195	0.314	3.27E-03	2.40E-09	1.64E-04	76.00
21	34270051	rs11700855	G	A	-0.0298	0.093	5.23E-03	1.20E-08	1.50E-04	69.56
22	24828853	rs71651683	T	C	-0.0705	0.014	1.28E-02	3.60E-08	1.39E-04	64.27

Table S16. Details of SNPs used for MR analysis of smoking initiation on HOA.

chr	pos	SNP	effect_allele	other_allele	beta	eaf	se	pval	R^2	F
1	44037685	rs3001723	A	G	0.0335	0.321	3.90E-03	8.12E-18	4.90E-04	309.94
1	73766037	rs7555507	T	C	-0.0241	0.496	3.56E-03	1.14E-11	2.91E-04	184.49
1	87905828	rs2050586	C	G	-0.0205	0.355	3.71E-03	3.00E-08	1.93E-04	122.36
1	154205120	rs12025237	C	A	-0.0330	0.124	5.34E-03	6.52E-10	2.37E-04	149.74
1	210304319	rs2046850	T	C	-0.0248	0.187	4.48E-03	3.03E-08	1.87E-04	118.49
2	45143175	rs578584	T	A	0.0287	0.605	3.60E-03	1.50E-15	3.93E-04	248.87
2	137542847	rs35702515	T	G	0.0252	0.162	4.23E-03	2.43E-09	1.73E-04	109.51
2	146143090	rs13030994	A	G	0.0361	0.485	3.56E-03	3.56E-24	6.51E-04	412.06
2	155682556	rs1445649	C	T	0.0240	0.525	3.56E-03	1.68E-11	2.87E-04	181.74
2	162802993	rs12474587	T	G	0.0276	0.404	3.58E-03	1.25E-14	3.68E-04	232.78
2	182034448	rs6433897	C	T	0.0224	0.754	4.06E-03	3.16E-08	1.87E-04	118.32
2	200937901	rs2107300	G	C	-0.0272	0.845	4.93E-03	3.27E-08	1.94E-04	122.67
2	226332033	rs4674993	G	A	-0.0252	0.207	4.44E-03	1.32E-08	2.09E-04	132.08

3	5724536	rs1869243	C	T	0.0197	0.481	3.56E-03	2.97E-08	1.95E-04	123.15
3	75009019	rs11712680	C	A	-0.0270	0.174	4.58E-03	3.51E-09	2.10E-04	133.10
3	117804154	rs1154693	G	A	0.0326	0.856	4.91E-03	3.12E-11	2.62E-04	166.06
4	28473524	rs962625	G	A	0.0237	0.24	4.04E-03	4.37E-09	2.05E-04	129.89
4	147797214	rs10001365	A	G	-0.0250	0.405	3.64E-03	6.65E-12	3.01E-04	190.54
5	60374912	rs6893752	G	A	-0.0241	0.766	4.07E-03	3.25E-09	2.08E-04	131.78
5	87756821	rs4352629	T	C	-0.0275	0.492	3.57E-03	1.22E-14	3.79E-04	239.80
5	103816655	rs12186738	T	G	-0.0333	0.154	5.02E-03	3.42E-11	2.88E-04	182.50
5	106834363	rs72789632	T	C	-0.0329	0.12	5.29E-03	5.02E-10	2.28E-04	144.57
5	154839646	rs1385108	T	C	0.0247	0.239	4.16E-03	3.00E-09	2.21E-04	140.03
5	166989513	rs4044321	G	A	-0.0278	0.642	3.71E-03	6.08E-14	3.56E-04	225.56
6	52916062	rs222449	T	A	-0.0253	0.793	4.43E-03	1.08E-08	2.10E-04	133.23
6	67405337	rs10498846	T	C	0.0206	0.473	3.56E-03	6.62E-09	2.12E-04	134.04
6	98748008	rs9401770	A	G	0.0277	0.273	3.99E-03	3.47E-12	3.05E-04	193.22
7	1708080	rs4236259	G	T	-0.0248	0.499	3.56E-03	3.35E-12	3.07E-04	194.17
7	1889773	rs10260968	A	G	-0.0203	0.597	3.61E-03	1.75E-08	1.99E-04	125.77
7	3407568	rs2140114	T	C	-0.0233	0.518	3.73E-03	4.70E-10	2.70E-04	170.99
7	96638267	rs3801289	C	A	-0.0221	0.351	3.74E-03	3.74E-09	2.22E-04	140.35
7	117523709	rs10233018	G	A	0.0271	0.503	3.56E-03	2.75E-14	3.66E-04	231.91
7	133589846	rs10279261	A	G	-0.0214	0.619	3.66E-03	5.00E-09	2.16E-04	136.97
8	27426077	rs1565735	A	T	-0.0376	0.212	4.46E-03	3.42E-17	4.73E-04	299.33
8	59814666	rs13261666	T	G	-0.0269	0.522	3.56E-03	3.90E-14	3.61E-04	228.50
8	65073605	rs12545053	G	A	0.0203	0.397	3.64E-03	2.43E-08	1.97E-04	124.64
8	91995577	rs2631024	G	A	-0.0230	0.737	4.03E-03	1.18E-08	2.04E-04	129.40
8	93201036	rs1899896	T	C	0.0264	0.286	3.89E-03	1.04E-11	2.86E-04	180.83
9	3014254	rs4543592	C	T	0.0219	0.468	3.56E-03	7.46E-10	2.40E-04	151.60
9	11070165	rs10114490	A	G	-0.0255	0.198	4.53E-03	1.81E-08	2.07E-04	130.86

9	86707289	rs2378662	A	G	0.0209	0.556	3.57E-03	4.16E-09	2.17E-04	137.13
10	8803551	rs10905461	C	T	-0.0240	0.718	4.15E-03	7.35E-09	2.32E-04	147.09
10	104563808	rs12356821	C	G	0.0394	0.14	5.05E-03	6.27E-15	3.73E-04	236.27
10	125680419	rs9423279	G	C	-0.0205	0.641	3.71E-03	3.21E-08	1.94E-04	122.57
11	7950797	rs4523689	G	A	-0.0206	0.408	3.64E-03	1.55E-08	2.05E-04	129.86
11	85980958	rs7929518	G	A	0.0242	0.765	4.28E-03	1.56E-08	2.11E-04	133.69
12	16748721	rs11057005	G	A	-0.0209	0.43	3.58E-03	4.85E-09	2.15E-04	135.91
12	121389500	rs1971318	T	C	0.0285	0.141	4.93E-03	7.06E-09	1.97E-04	124.60
13	38357471	rs3904512	A	G	-0.0212	0.429	3.58E-03	3.23E-09	2.19E-04	138.83
13	100548329	rs7322872	T	C	-0.0256	0.782	4.33E-03	3.58E-09	2.23E-04	141.11
14	29500130	rs76214862	C	A	-0.0250	0.202	4.55E-03	3.99E-08	2.01E-04	127.43
15	83922387	rs12441907	A	C	-0.0292	0.186	4.52E-03	1.06E-10	2.58E-04	163.48
16	717085	rs7197072	T	C	-0.0248	0.238	4.17E-03	2.77E-09	2.22E-04	140.82
16	17572674	rs4781977	C	T	-0.0239	0.205	4.36E-03	4.54E-08	1.86E-04	117.51
16	65604652	rs4785836	C	T	-0.0205	0.398	3.66E-03	2.26E-08	2.01E-04	127.09
16	87443734	rs1050847	T	C	-0.0216	0.505	3.59E-03	1.67E-09	2.34E-04	147.96
17	2072949	rs11658881	G	A	0.0201	0.418	3.61E-03	2.43E-08	1.97E-04	124.86
17	7795972	rs11078713	G	A	-0.0202	0.454	3.61E-03	2.23E-08	2.02E-04	127.68
17	30657058	rs7224742	T	C	-0.0207	0.595	3.66E-03	1.43E-08	2.07E-04	130.83
18	42632652	rs72896886	C	G	-0.0269	0.144	4.84E-03	2.75E-08	1.78E-04	112.81
18	72535282	rs11872397	A	G	-0.0248	0.252	4.09E-03	1.43E-09	2.31E-04	146.43
19	4474725	rs76608582	A	C	-0.0496	0.0389	8.26E-03	1.94E-09	1.84E-04	116.23
20	31175258	rs1555445	T	A	0.0226	0.337	3.82E-03	3.65E-09	2.27E-04	143.89
21	40555561	rs117143374	C	T	0.0293	0.12	5.27E-03	2.76E-08	1.81E-04	114.68
22	28781758	rs134529	C	T	-0.0200	0.349	3.66E-03	4.85E-08	1.81E-04	114.85

Table S17. Details of SNPs used for MR analysis of smoking initiation on KOA.

chr	pos	SNP	effect_allele	other_allele	beta	eaf	se	pval	R^2	F
1	44037685	rs3001723	A	G	0.0335	0.321	3.90E-03	8.12E-18	4.90E-04	309.94
1	73766037	rs7555507	T	C	-0.0241	0.496	3.56E-03	1.14E-11	2.91E-04	184.49
1	87905828	rs2050586	C	G	-0.0205	0.355	3.71E-03	3.00E-08	1.93E-04	122.36
1	154205120	rs12025237	C	A	-0.0330	0.124	5.34E-03	6.52E-10	2.37E-04	149.74
1	210304319	rs2046850	T	C	-0.0248	0.187	4.48E-03	3.03E-08	1.87E-04	118.49
2	45143175	rs578584	T	A	0.0287	0.605	3.60E-03	1.50E-15	3.93E-04	248.87
2	137542847	rs35702515	T	G	0.0252	0.162	4.23E-03	2.43E-09	1.73E-04	109.51
2	146143090	rs13030994	A	G	0.0361	0.485	3.56E-03	3.56E-24	6.51E-04	412.06
2	155682556	rs1445649	C	T	0.0240	0.525	3.56E-03	1.68E-11	2.87E-04	181.74
2	162802993	rs12474587	T	G	0.0276	0.404	3.58E-03	1.25E-14	3.68E-04	232.78
2	182034448	rs6433897	C	T	0.0224	0.754	4.06E-03	3.16E-08	1.87E-04	118.32
2	200937901	rs2107300	G	C	-0.0272	0.845	4.93E-03	3.27E-08	1.94E-04	122.67
2	226332033	rs4674993	G	A	-0.0252	0.207	4.44E-03	1.32E-08	2.09E-04	132.08
3	5724536	rs1869243	C	T	0.0197	0.481	3.56E-03	2.97E-08	1.95E-04	123.15
3	75009019	rs11712680	C	A	-0.0270	0.174	4.58E-03	3.51E-09	2.10E-04	133.10
3	117804154	rs1154693	G	A	0.0326	0.856	4.91E-03	3.12E-11	2.62E-04	166.06
4	28473524	rs962625	G	A	0.0237	0.24	4.04E-03	4.37E-09	2.05E-04	129.89
4	147797214	rs10001365	A	G	-0.0250	0.405	3.64E-03	6.65E-12	3.01E-04	190.54
5	60374912	rs6893752	G	A	-0.0241	0.766	4.07E-03	3.25E-09	2.08E-04	131.78
5	87756821	rs4352629	T	C	-0.0275	0.492	3.57E-03	1.22E-14	3.79E-04	239.80
5	103816655	rs12186738	T	G	-0.0333	0.154	5.02E-03	3.42E-11	2.88E-04	182.50
5	106834363	rs72789632	T	C	-0.0329	0.12	5.29E-03	5.02E-10	2.28E-04	144.57
5	154839646	rs1385108	T	C	0.0247	0.239	4.16E-03	3.00E-09	2.21E-04	140.03
5	166989513	rs4044321	G	A	-0.0278	0.642	3.71E-03	6.08E-14	3.56E-04	225.56
6	52916062	rs222449	T	A	-0.0253	0.793	4.43E-03	1.08E-08	2.10E-04	133.23

6	67405337	rs10498846	T	C	0.0206	0.473	3.56E-03	6.62E-09	2.12E-04	134.04
6	98748008	rs9401770	A	G	0.0277	0.273	3.99E-03	3.47E-12	3.05E-04	193.22
7	1708080	rs4236259	G	T	-0.0248	0.499	3.56E-03	3.35E-12	3.07E-04	194.17
7	3407568	rs2140114	T	C	-0.0233	0.518	3.73E-03	4.70E-10	2.70E-04	170.99
7	117523709	rs10233018	G	A	0.0271	0.503	3.56E-03	2.75E-14	3.66E-04	231.91
7	133589846	rs10279261	A	G	-0.0214	0.619	3.66E-03	5.00E-09	2.16E-04	136.97
8	27426077	rs1565735	A	T	-0.0376	0.212	4.46E-03	3.42E-17	4.73E-04	299.33
8	59814666	rs13261666	T	G	-0.0269	0.522	3.56E-03	3.90E-14	3.61E-04	228.50
8	65073605	rs12545053	G	A	0.0203	0.397	3.64E-03	2.43E-08	1.97E-04	124.64
8	91995577	rs2631024	G	A	-0.0230	0.737	4.03E-03	1.18E-08	2.04E-04	129.40
8	93201036	rs1899896	T	C	0.0264	0.286	3.89E-03	1.04E-11	2.86E-04	180.83
9	3014254	rs4543592	C	T	0.0219	0.468	3.56E-03	7.46E-10	2.40E-04	151.60
9	11070165	rs10114490	A	G	-0.0255	0.198	4.53E-03	1.81E-08	2.07E-04	130.86
9	86707289	rs2378662	A	G	0.0209	0.556	3.57E-03	4.16E-09	2.17E-04	137.13
10	8803551	rs10905461	C	T	-0.0240	0.718	4.15E-03	7.35E-09	2.32E-04	147.09
10	104563808	rs12356821	C	G	0.0394	0.14	5.05E-03	6.27E-15	3.73E-04	236.27
10	125680419	rs9423279	G	C	-0.0205	0.641	3.71E-03	3.21E-08	1.94E-04	122.57
11	7950797	rs4523689	G	A	-0.0206	0.408	3.64E-03	1.55E-08	2.05E-04	129.86
11	85980958	rs7929518	G	A	0.0242	0.765	4.28E-03	1.56E-08	2.11E-04	133.69
12	16748721	rs11057005	G	A	-0.0209	0.43	3.58E-03	4.85E-09	2.15E-04	135.91
12	121389500	rs1971318	T	C	0.0285	0.141	4.93E-03	7.06E-09	1.97E-04	124.60
13	38357471	rs3904512	A	G	-0.0212	0.429	3.58E-03	3.23E-09	2.19E-04	138.83
14	29500130	rs76214862	C	A	-0.0250	0.202	4.55E-03	3.99E-08	2.01E-04	127.43
15	83922387	rs12441907	A	C	-0.0292	0.186	4.52E-03	1.06E-10	2.58E-04	163.48
16	717085	rs7197072	T	C	-0.0248	0.238	4.17E-03	2.77E-09	2.22E-04	140.82
16	17572674	rs4781977	C	T	-0.0239	0.205	4.36E-03	4.54E-08	1.86E-04	117.51

16	65604652	rs4785836	C	T	-0.0205	0.398	3.66E-03	2.26E-08	2.01E-04	127.09
16	87443734	rs1050847	T	C	-0.0216	0.505	3.59E-03	1.67E-09	2.34E-04	147.96
17	2072949	rs11658881	G	A	0.0201	0.418	3.61E-03	2.43E-08	1.97E-04	124.86
17	7795972	rs11078713	G	A	-0.0202	0.454	3.61E-03	2.23E-08	2.02E-04	127.68
17	30657058	rs7224742	T	C	-0.0207	0.595	3.66E-03	1.43E-08	2.07E-04	130.83
18	42632652	rs72896886	C	G	-0.0269	0.144	4.84E-03	2.75E-08	1.78E-04	112.81
18	72535282	rs11872397	A	G	-0.0248	0.252	4.09E-03	1.43E-09	2.31E-04	146.43
19	4474725	rs76608582	A	C	-0.0496	0.0389	8.26E-03	1.94E-09	1.84E-04	116.23
20	31175258	rs1555445	T	A	0.0226	0.337	3.82E-03	3.65E-09	2.27E-04	143.89
21	40555561	rs117143374	C	T	0.0293	0.12	5.27E-03	2.76E-08	1.81E-04	114.68
22	28781758	rs134529	C	T	-0.0200	0.349	3.66E-03	4.85E-08	1.81E-04	114.85

Table S18. Details of SNPs used for MR analysis of smoking initiation on RA(M13).

chr	pos	SNP	effect_allele	other_allele	beta	eaf	se	pval	R^2	F
1	8481016	rs301805	G	T	0.0215	0.559	3.61E-03	2.80E-09	2.27E-04	143.82
1	44037685	rs3001723	A	G	0.0335	0.321	3.90E-03	8.12E-18	4.90E-04	309.94
1	50625979	rs6669839	T	C	0.0260	0.204	4.40E-03	3.36E-09	2.20E-04	139.00
1	73766037	rs7555507	T	C	-0.0241	0.496	3.56E-03	1.14E-11	2.91E-04	184.49
1	87905828	rs2050586	C	G	-0.0205	0.355	3.71E-03	3.00E-08	1.93E-04	122.36
1	91196176	rs12042107	C	T	-0.0223	0.527	3.57E-03	4.22E-10	2.48E-04	156.69
1	154205120	rs12025237	C	A	-0.0330	0.124	5.34E-03	6.52E-10	2.37E-04	149.74
1	210304319	rs2046850	T	C	-0.0248	0.187	4.48E-03	3.03E-08	1.87E-04	118.49
2	623976	rs6728726	C	T	0.0354	0.829	4.73E-03	6.73E-14	3.56E-04	225.53
2	45143175	rs578584	T	A	0.0287	0.605	3.60E-03	1.50E-15	3.93E-04	248.87
2	104088751	rs266047	A	G	-0.0305	0.529	3.74E-03	3.36E-16	4.64E-04	293.67
2	137542847	rs35702515	T	G	0.0252	0.162	4.23E-03	2.43E-09	1.73E-04	109.51
2	146143090	rs13030994	A	G	0.0361	0.485	3.56E-03	3.56E-24	6.51E-04	412.06

2	155682556	rs1445649	C	T	0.0240	0.525	3.56E-03	1.68E-11	2.87E-04	181.74
2	162802993	rs12474587	T	G	0.0276	0.404	3.58E-03	1.25E-14	3.68E-04	232.78
2	182034448	rs6433897	C	T	0.0224	0.754	4.06E-03	3.16E-08	1.87E-04	118.32
2	200937901	rs2107300	G	C	-0.0272	0.845	4.93E-03	3.27E-08	1.94E-04	122.67
2	226332033	rs4674993	G	A	-0.0252	0.207	4.44E-03	1.32E-08	2.09E-04	132.08
3	5724536	rs1869243	C	T	0.0197	0.481	3.56E-03	2.97E-08	1.95E-04	123.15
3	50224225	rs12632110	G	A	-0.0234	0.647	3.75E-03	4.78E-10	2.50E-04	158.00
3	75009019	rs11712680	C	A	-0.0270	0.174	4.58E-03	3.51E-09	2.10E-04	133.10
3	85624131	rs6788098	T	A	-0.0313	0.623	3.69E-03	1.91E-17	4.62E-04	292.21
3	85766025	rs9835772	T	A	0.0240	0.235	4.14E-03	6.32E-09	2.08E-04	131.60
3	85985324	rs66680800	T	G	-0.0203	0.397	3.65E-03	2.83E-08	1.97E-04	124.53
3	117804154	rs1154693	G	A	0.0326	0.856	4.91E-03	3.12E-11	2.62E-04	166.06
4	28473524	rs962625	G	A	0.0237	0.24	4.04E-03	4.37E-09	2.05E-04	129.89
4	67825894	rs993700	C	T	-0.0259	0.766	4.29E-03	1.53E-09	2.41E-04	152.54
4	140927812	rs13145728	C	G	-0.0233	0.358	3.66E-03	2.14E-10	2.49E-04	157.29
4	147797214	rs10001365	A	G	-0.0250	0.405	3.64E-03	6.65E-12	3.01E-04	190.54
5	60374912	rs6893752	G	A	-0.0241	0.766	4.07E-03	3.25E-09	2.08E-04	131.78
5	87756821	rs4352629	T	C	-0.0275	0.492	3.57E-03	1.22E-14	3.79E-04	239.80
5	103816655	rs12186738	T	G	-0.0333	0.154	5.02E-03	3.42E-11	2.88E-04	182.50
5	106834363	rs72789632	T	C	-0.0329	0.12	5.29E-03	5.02E-10	2.28E-04	144.57
5	154839646	rs1385108	T	C	0.0247	0.239	4.16E-03	3.00E-09	2.21E-04	140.03
5	166989513	rs4044321	G	A	-0.0278	0.642	3.71E-03	6.08E-14	3.56E-04	225.56
6	52916062	rs222449	T	A	-0.0253	0.793	4.43E-03	1.08E-08	2.10E-04	133.23
6	67405337	rs10498846	T	C	0.0206	0.473	3.56E-03	6.62E-09	2.12E-04	134.04
6	98748008	rs9401770	A	G	0.0277	0.273	3.99E-03	3.47E-12	3.05E-04	193.22
6	108994161	rs3800227	G	A	0.0228	0.701	4.06E-03	1.93E-08	2.18E-04	138.07

6	111644332	rs240963	C	T	-0.0410	0.836	4.84E-03	2.16E-17	4.62E-04	292.45
7	1708080	rs4236259	G	T	-0.0248	0.499	3.56E-03	3.35E-12	3.07E-04	194.17
7	1889773	rs10260968	A	G	-0.0203	0.597	3.61E-03	1.75E-08	1.99E-04	125.77
7	3407568	rs2140114	T	C	-0.0233	0.518	3.73E-03	4.70E-10	2.70E-04	170.99
7	69735251	rs12112638	G	A	-0.0245	0.275	4.04E-03	1.34E-09	2.40E-04	151.82
7	96638267	rs3801289	C	A	-0.0221	0.351	3.74E-03	3.74E-09	2.22E-04	140.35
7	99185406	rs12333760	C	T	-0.0290	0.204	4.80E-03	1.44E-09	2.74E-04	173.44
7	117523709	rs10233018	G	A	0.0271	0.503	3.56E-03	2.75E-14	3.66E-04	231.91
7	133589846	rs10279261	A	G	-0.0214	0.619	3.66E-03	5.00E-09	2.16E-04	136.97
8	27426077	rs1565735	A	T	-0.0376	0.212	4.46E-03	3.42E-17	4.73E-04	299.33
8	59814666	rs13261666	T	G	-0.0269	0.522	3.56E-03	3.90E-14	3.61E-04	228.50
8	65073605	rs12545053	G	A	0.0203	0.397	3.64E-03	2.43E-08	1.97E-04	124.64
8	91995577	rs2631024	G	A	-0.0230	0.737	4.03E-03	1.18E-08	2.04E-04	129.40
8	93201036	rs1899896	T	C	0.0264	0.286	3.89E-03	1.04E-11	2.86E-04	180.83
9	3014254	rs4543592	C	T	0.0219	0.468	3.56E-03	7.46E-10	2.40E-04	151.60
9	86707289	rs2378662	A	G	0.0209	0.556	3.57E-03	4.16E-09	2.17E-04	137.13
10	8803551	rs10905461	C	T	-0.0240	0.718	4.15E-03	7.35E-09	2.32E-04	147.09
10	21766969	rs10159545	G	C	0.0263	0.375	3.73E-03	1.84E-12	3.23E-04	204.46
10	104563808	rs12356821	C	G	0.0394	0.14	5.05E-03	6.27E-15	3.73E-04	236.27
10	125680419	rs9423279	G	C	-0.0205	0.641	3.71E-03	3.21E-08	1.94E-04	122.57
11	7950797	rs4523689	G	A	-0.0206	0.408	3.64E-03	1.55E-08	2.05E-04	129.86
11	27679916	rs6265	T	C	-0.0318	0.203	4.58E-03	3.77E-12	3.27E-04	206.95
11	85980958	rs7929518	G	A	0.0242	0.765	4.28E-03	1.56E-08	2.11E-04	133.69
11	112911004	rs7938812	G	T	0.0438	0.424	3.64E-03	2.71E-33	9.37E-04	593.29
12	16748721	rs11057005	G	A	-0.0209	0.43	3.58E-03	4.85E-09	2.15E-04	135.91
12	56508409	rs4759228	C	G	-0.0217	0.27	3.93E-03	3.58E-08	1.85E-04	117.39
12	69655167	rs7969559	G	A	-0.0244	0.688	3.96E-03	7.31E-10	2.55E-04	161.46

12	121389500	rs1971318	T	C	0.0285	0.141	4.93E-03	7.06E-09	1.97E-04	124.60
13	38357471	rs3904512	A	G	-0.0212	0.429	3.58E-03	3.23E-09	2.19E-04	138.83
14	29500130	rs76214862	C	A	-0.0250	0.202	4.55E-03	3.99E-08	2.01E-04	127.43
15	47935843	rs1435741	A	G	0.0294	0.425	3.59E-03	2.64E-16	4.23E-04	267.72
15	83922387	rs12441907	A	C	-0.0292	0.186	4.52E-03	1.06E-10	2.58E-04	163.48
16	717085	rs7197072	T	C	-0.0248	0.238	4.17E-03	2.77E-09	2.22E-04	140.82
16	17572674	rs4781977	C	T	-0.0239	0.205	4.36E-03	4.54E-08	1.86E-04	117.51
16	65604652	rs4785836	C	T	-0.0205	0.398	3.66E-03	2.26E-08	2.01E-04	127.09
16	87443734	rs1050847	T	C	-0.0216	0.505	3.59E-03	1.67E-09	2.34E-04	147.96
17	2072949	rs11658881	G	A	0.0201	0.418	3.61E-03	2.43E-08	1.97E-04	124.86
17	7795972	rs11078713	G	A	-0.0202	0.454	3.61E-03	2.23E-08	2.02E-04	127.68
17	30657058	rs7224742	T	C	-0.0207	0.595	3.66E-03	1.43E-08	2.07E-04	130.83
18	42632652	rs72896886	C	G	-0.0269	0.144	4.84E-03	2.75E-08	1.78E-04	112.81
18	72535282	rs11872397	A	G	-0.0248	0.252	4.09E-03	1.43E-09	2.31E-04	146.43
19	4474725	rs76608582	A	C	-0.0496	0.0389	8.26E-03	1.94E-09	1.84E-04	116.23
21	40555561	rs117143374	C	T	0.0293	0.12	5.27E-03	2.76E-08	1.81E-04	114.68
22	28781758	rs134529	C	T	-0.0200	0.349	3.66E-03	4.85E-08	1.81E-04	114.85

Table S19. Details of SNPs used for MR analysis of smoking initiation on Seronegative RA.

chr	pos	SNP	effect_allele	other_allele	beta	eaf	se	pval	R^2	F
1	8481016	rs301805	G	T	0.0215	0.559	3.61E-03	2.80E-09	2.27E-04	143.82
1	44037685	rs3001723	A	G	0.0335	0.321	3.90E-03	8.12E-18	4.90E-04	309.94
1	50625979	rs6669839	T	C	0.0260	0.204	4.40E-03	3.36E-09	2.20E-04	139.00
1	73766037	rs7555507	T	C	-0.0241	0.496	3.56E-03	1.14E-11	2.91E-04	184.49
1	87905828	rs2050586	C	G	-0.0205	0.355	3.71E-03	3.00E-08	1.93E-04	122.36
1	91196176	rs12042107	C	T	-0.0223	0.527	3.57E-03	4.22E-10	2.48E-04	156.69

1	154205120	rs12025237	C	A	-0.0330	0.124	5.34E-03	6.52E-10	2.37E-04	149.74
1	210304319	rs2046850	T	C	-0.0248	0.187	4.48E-03	3.03E-08	1.87E-04	118.49
2	623976	rs6728726	C	T	0.0354	0.829	4.73E-03	6.73E-14	3.56E-04	225.53
2	45143175	rs578584	T	A	0.0287	0.605	3.60E-03	1.50E-15	3.93E-04	248.87
2	104088751	rs266047	A	G	-0.0305	0.529	3.74E-03	3.36E-16	4.64E-04	293.67
2	137542847	rs35702515	T	G	0.0252	0.162	4.23E-03	2.43E-09	1.73E-04	109.51
2	146143090	rs13030994	A	G	0.0361	0.485	3.56E-03	3.56E-24	6.51E-04	412.06
2	155682556	rs1445649	C	T	0.0240	0.525	3.56E-03	1.68E-11	2.87E-04	181.74
2	162802993	rs12474587	T	G	0.0276	0.404	3.58E-03	1.25E-14	3.68E-04	232.78
2	182034448	rs6433897	C	T	0.0224	0.754	4.06E-03	3.16E-08	1.87E-04	118.32
2	200937901	rs2107300	G	C	-0.0272	0.845	4.93E-03	3.27E-08	1.94E-04	122.67
2	226332033	rs4674993	G	A	-0.0252	0.207	4.44E-03	1.32E-08	2.09E-04	132.08
3	5724536	rs1869243	C	T	0.0197	0.481	3.56E-03	2.97E-08	1.95E-04	123.15
3	50224225	rs12632110	G	A	-0.0234	0.647	3.75E-03	4.78E-10	2.50E-04	158.00
3	75009019	rs11712680	C	A	-0.0270	0.174	4.58E-03	3.51E-09	2.10E-04	133.10
3	85624131	rs6788098	T	A	-0.0313	0.623	3.69E-03	1.91E-17	4.62E-04	292.21
3	85766025	rs9835772	T	A	0.0240	0.235	4.14E-03	6.32E-09	2.08E-04	131.60
3	85985324	rs66680800	T	G	-0.0203	0.397	3.65E-03	2.83E-08	1.97E-04	124.53
3	117804154	rs1154693	G	A	0.0326	0.856	4.91E-03	3.12E-11	2.62E-04	166.06
4	28473524	rs962625	G	A	0.0237	0.24	4.04E-03	4.37E-09	2.05E-04	129.89
4	67825894	rs993700	C	T	-0.0259	0.766	4.29E-03	1.53E-09	2.41E-04	152.54
4	140927812	rs13145728	C	G	-0.0233	0.358	3.66E-03	2.14E-10	2.49E-04	157.29
4	147797214	rs10001365	A	G	-0.0250	0.405	3.64E-03	6.65E-12	3.01E-04	190.54
5	60374912	rs6893752	G	A	-0.0241	0.766	4.07E-03	3.25E-09	2.08E-04	131.78
5	87756821	rs4352629	T	C	-0.0275	0.492	3.57E-03	1.22E-14	3.79E-04	239.80
5	103816655	rs12186738	T	G	-0.0333	0.154	5.02E-03	3.42E-11	2.88E-04	182.50
5	106834363	rs72789632	T	C	-0.0329	0.12	5.29E-03	5.02E-10	2.28E-04	144.57

5	154839646	rs1385108	T	C	0.0247	0.239	4.16E-03	3.00E-09	2.21E-04	140.03
5	166989513	rs4044321	G	A	-0.0278	0.642	3.71E-03	6.08E-14	3.56E-04	225.56
6	52916062	rs222449	T	A	-0.0253	0.793	4.43E-03	1.08E-08	2.10E-04	133.23
6	67405337	rs10498846	T	C	0.0206	0.473	3.56E-03	6.62E-09	2.12E-04	134.04
6	98748008	rs9401770	A	G	0.0277	0.273	3.99E-03	3.47E-12	3.05E-04	193.22
6	108994161	rs3800227	G	A	0.0228	0.701	4.06E-03	1.93E-08	2.18E-04	138.07
6	111644332	rs240963	C	T	-0.0410	0.836	4.84E-03	2.16E-17	4.62E-04	292.45
7	1708080	rs4236259	G	T	-0.0248	0.499	3.56E-03	3.35E-12	3.07E-04	194.17
7	1889773	rs10260968	A	G	-0.0203	0.597	3.61E-03	1.75E-08	1.99E-04	125.77
7	3407568	rs2140114	T	C	-0.0233	0.518	3.73E-03	4.70E-10	2.70E-04	170.99
7	69735251	rs12112638	G	A	-0.0245	0.275	4.04E-03	1.34E-09	2.40E-04	151.82
7	96638267	rs3801289	C	A	-0.0221	0.351	3.74E-03	3.74E-09	2.22E-04	140.35
7	99185406	rs12333760	C	T	-0.0290	0.204	4.80E-03	1.44E-09	2.74E-04	173.44
7	117523709	rs10233018	G	A	0.0271	0.503	3.56E-03	2.75E-14	3.66E-04	231.91
7	133589846	rs10279261	A	G	-0.0214	0.619	3.66E-03	5.00E-09	2.16E-04	136.97
8	27426077	rs1565735	A	T	-0.0376	0.212	4.46E-03	3.42E-17	4.73E-04	299.33
8	59814666	rs13261666	T	G	-0.0269	0.522	3.56E-03	3.90E-14	3.61E-04	228.50
8	65073605	rs12545053	G	A	0.0203	0.397	3.64E-03	2.43E-08	1.97E-04	124.64
8	91995577	rs2631024	G	A	-0.0230	0.737	4.03E-03	1.18E-08	2.04E-04	129.40
8	93201036	rs1899896	T	C	0.0264	0.286	3.89E-03	1.04E-11	2.86E-04	180.83
9	3014254	rs4543592	C	T	0.0219	0.468	3.56E-03	7.46E-10	2.40E-04	151.60
9	86707289	rs2378662	A	G	0.0209	0.556	3.57E-03	4.16E-09	2.17E-04	137.13
10	8803551	rs10905461	C	T	-0.0240	0.718	4.15E-03	7.35E-09	2.32E-04	147.09
10	21766969	rs10159545	G	C	0.0263	0.375	3.73E-03	1.84E-12	3.23E-04	204.46
10	104563808	rs12356821	C	G	0.0394	0.14	5.05E-03	6.27E-15	3.73E-04	236.27
10	125680419	rs9423279	G	C	-0.0205	0.641	3.71E-03	3.21E-08	1.94E-04	122.57

11	7950797	rs4523689	G	A	-0.0206	0.408	3.64E-03	1.55E-08	2.05E-04	129.86
11	27679916	rs6265	T	C	-0.0318	0.203	4.58E-03	3.77E-12	3.27E-04	206.95
11	85980958	rs7929518	G	A	0.0242	0.765	4.28E-03	1.56E-08	2.11E-04	133.69
11	112911004	rs7938812	G	T	0.0438	0.424	3.64E-03	2.71E-33	9.37E-04	593.29
12	16748721	rs11057005	G	A	-0.0209	0.43	3.58E-03	4.85E-09	2.15E-04	135.91
12	56508409	rs4759228	C	G	-0.0217	0.27	3.93E-03	3.58E-08	1.85E-04	117.39
12	69655167	rs7969559	G	A	-0.0244	0.688	3.96E-03	7.31E-10	2.55E-04	161.46
12	121389500	rs1971318	T	C	0.0285	0.141	4.93E-03	7.06E-09	1.97E-04	124.60
13	38357471	rs3904512	A	G	-0.0212	0.429	3.58E-03	3.23E-09	2.19E-04	138.83
14	29500130	rs76214862	C	A	-0.0250	0.202	4.55E-03	3.99E-08	2.01E-04	127.43
15	47935843	rs1435741	A	G	0.0294	0.425	3.59E-03	2.64E-16	4.23E-04	267.72
15	83922387	rs12441907	A	C	-0.0292	0.186	4.52E-03	1.06E-10	2.58E-04	163.48
16	717085	rs7197072	T	C	-0.0248	0.238	4.17E-03	2.77E-09	2.22E-04	140.82
16	17572674	rs4781977	C	T	-0.0239	0.205	4.36E-03	4.54E-08	1.86E-04	117.51
16	65604652	rs4785836	C	T	-0.0205	0.398	3.66E-03	2.26E-08	2.01E-04	127.09
16	87443734	rs1050847	T	C	-0.0216	0.505	3.59E-03	1.67E-09	2.34E-04	147.96
17	2072949	rs11658881	G	A	0.0201	0.418	3.61E-03	2.43E-08	1.97E-04	124.86
17	7795972	rs11078713	G	A	-0.0202	0.454	3.61E-03	2.23E-08	2.02E-04	127.68
17	30657058	rs7224742	T	C	-0.0207	0.595	3.66E-03	1.43E-08	2.07E-04	130.83
18	42632652	rs72896886	C	G	-0.0269	0.144	4.84E-03	2.75E-08	1.78E-04	112.81
18	72535282	rs11872397	A	G	-0.0248	0.252	4.09E-03	1.43E-09	2.31E-04	146.43
19	4474725	rs76608582	A	C	-0.0496	0.0389	8.26E-03	1.94E-09	1.84E-04	116.23
21	40555561	rs117143374	C	T	0.0293	0.12	5.27E-03	2.76E-08	1.81E-04	114.68
22	28781758	rs134529	C	T	-0.0200	0.349	3.66E-03	4.85E-08	1.81E-04	114.85

Table S20. Details of SNPs used for MR analysis of smoking initiation on Seropositive RA.

chr	pos	SNP	effect_allele	other_allele	beta	eaf	se	pval	R^2	F

1	8481016	rs301805	G	T	0.0215	0.559	3.61E-03	2.80E-09	2.27E-04	143.82
1	44037685	rs3001723	A	G	0.0335	0.321	3.90E-03	8.12E-18	4.90E-04	309.94
1	50625979	rs6669839	T	C	0.0260	0.204	4.40E-03	3.36E-09	2.20E-04	139.00
1	73766037	rs7555507	T	C	-0.0241	0.496	3.56E-03	1.14E-11	2.91E-04	184.49
1	87905828	rs2050586	C	G	-0.0205	0.355	3.71E-03	3.00E-08	1.93E-04	122.36
1	91196176	rs12042107	C	T	-0.0223	0.527	3.57E-03	4.22E-10	2.48E-04	156.69
1	154205120	rs12025237	C	A	-0.0330	0.124	5.34E-03	6.52E-10	2.37E-04	149.74
1	210304319	rs2046850	T	C	-0.0248	0.187	4.48E-03	3.03E-08	1.87E-04	118.49
2	623976	rs6728726	C	T	0.0354	0.829	4.73E-03	6.73E-14	3.56E-04	225.53
2	45143175	rs578584	T	A	0.0287	0.605	3.60E-03	1.50E-15	3.93E-04	248.87
2	104088751	rs266047	A	G	-0.0305	0.529	3.74E-03	3.36E-16	4.64E-04	293.67
2	137542847	rs35702515	T	G	0.0252	0.162	4.23E-03	2.43E-09	1.73E-04	109.51
2	146143090	rs13030994	A	G	0.0361	0.485	3.56E-03	3.56E-24	6.51E-04	412.06
2	155682556	rs1445649	C	T	0.0240	0.525	3.56E-03	1.68E-11	2.87E-04	181.74
2	162802993	rs12474587	T	G	0.0276	0.404	3.58E-03	1.25E-14	3.68E-04	232.78
2	182034448	rs6433897	C	T	0.0224	0.754	4.06E-03	3.16E-08	1.87E-04	118.32
2	200937901	rs2107300	G	C	-0.0272	0.845	4.93E-03	3.27E-08	1.94E-04	122.67
2	226332033	rs4674993	G	A	-0.0252	0.207	4.44E-03	1.32E-08	2.09E-04	132.08
3	5724536	rs1869243	C	T	0.0197	0.481	3.56E-03	2.97E-08	1.95E-04	123.15
3	50224225	rs12632110	G	A	-0.0234	0.647	3.75E-03	4.78E-10	2.50E-04	158.00
3	75009019	rs11712680	C	A	-0.0270	0.174	4.58E-03	3.51E-09	2.10E-04	133.10
3	85624131	rs6788098	T	A	-0.0313	0.623	3.69E-03	1.91E-17	4.62E-04	292.21
3	85766025	rs9835772	T	A	0.0240	0.235	4.14E-03	6.32E-09	2.08E-04	131.60
3	85985324	rs66680800	T	G	-0.0203	0.397	3.65E-03	2.83E-08	1.97E-04	124.53
3	117804154	rs1154693	G	A	0.0326	0.856	4.91E-03	3.12E-11	2.62E-04	166.06
4	28473524	rs962625	G	A	0.0237	0.24	4.04E-03	4.37E-09	2.05E-04	129.89

4	67825894	rs993700	C	T	-0.0259	0.766	4.29E-03	1.53E-09	2.41E-04	152.54
4	140927812	rs13145728	C	G	-0.0233	0.358	3.66E-03	2.14E-10	2.49E-04	157.29
4	147797214	rs10001365	A	G	-0.0250	0.405	3.64E-03	6.65E-12	3.01E-04	190.54
5	60374912	rs6893752	G	A	-0.0241	0.766	4.07E-03	3.25E-09	2.08E-04	131.78
5	87756821	rs4352629	T	C	-0.0275	0.492	3.57E-03	1.22E-14	3.79E-04	239.80
5	103816655	rs12186738	T	G	-0.0333	0.154	5.02E-03	3.42E-11	2.88E-04	182.50
5	106834363	rs72789632	T	C	-0.0329	0.12	5.29E-03	5.02E-10	2.28E-04	144.57
5	154839646	rs1385108	T	C	0.0247	0.239	4.16E-03	3.00E-09	2.21E-04	140.03
5	166989513	rs4044321	G	A	-0.0278	0.642	3.71E-03	6.08E-14	3.56E-04	225.56
6	52916062	rs222449	T	A	-0.0253	0.793	4.43E-03	1.08E-08	2.10E-04	133.23
6	67405337	rs10498846	T	C	0.0206	0.473	3.56E-03	6.62E-09	2.12E-04	134.04
6	98748008	rs9401770	A	G	0.0277	0.273	3.99E-03	3.47E-12	3.05E-04	193.22
6	108994161	rs3800227	G	A	0.0228	0.701	4.06E-03	1.93E-08	2.18E-04	138.07
6	111644332	rs240963	C	T	-0.0410	0.836	4.84E-03	2.16E-17	4.62E-04	292.45
7	1708080	rs4236259	G	T	-0.0248	0.499	3.56E-03	3.35E-12	3.07E-04	194.17
7	1889773	rs10260968	A	G	-0.0203	0.597	3.61E-03	1.75E-08	1.99E-04	125.77
7	3407568	rs2140114	T	C	-0.0233	0.518	3.73E-03	4.70E-10	2.70E-04	170.99
7	69735251	rs12112638	G	A	-0.0245	0.275	4.04E-03	1.34E-09	2.40E-04	151.82
7	96638267	rs3801289	C	A	-0.0221	0.351	3.74E-03	3.74E-09	2.22E-04	140.35
7	99185406	rs12333760	C	T	-0.0290	0.204	4.80E-03	1.44E-09	2.74E-04	173.44
7	117523709	rs10233018	G	A	0.0271	0.503	3.56E-03	2.75E-14	3.66E-04	231.91
7	133589846	rs10279261	A	G	-0.0214	0.619	3.66E-03	5.00E-09	2.16E-04	136.97
8	27426077	rs1565735	A	T	-0.0376	0.212	4.46E-03	3.42E-17	4.73E-04	299.33
8	59814666	rs13261666	T	G	-0.0269	0.522	3.56E-03	3.90E-14	3.61E-04	228.50
8	65073605	rs12545053	G	A	0.0203	0.397	3.64E-03	2.43E-08	1.97E-04	124.64
8	91995577	rs2631024	G	A	-0.0230	0.737	4.03E-03	1.18E-08	2.04E-04	129.40
8	93201036	rs1899896	T	C	0.0264	0.286	3.89E-03	1.04E-11	2.86E-04	180.83

9	3014254	rs4543592	C	T	0.0219	0.468	3.56E-03	7.46E-10	2.40E-04	151.60
9	86707289	rs2378662	A	G	0.0209	0.556	3.57E-03	4.16E-09	2.17E-04	137.13
10	8803551	rs10905461	C	T	-0.0240	0.718	4.15E-03	7.35E-09	2.32E-04	147.09
10	21766969	rs10159545	G	C	0.0263	0.375	3.73E-03	1.84E-12	3.23E-04	204.46
10	104563808	rs12356821	C	G	0.0394	0.14	5.05E-03	6.27E-15	3.73E-04	236.27
10	125680419	rs9423279	G	C	-0.0205	0.641	3.71E-03	3.21E-08	1.94E-04	122.57
11	7950797	rs4523689	G	A	-0.0206	0.408	3.64E-03	1.55E-08	2.05E-04	129.86
11	27679916	rs6265	T	C	-0.0318	0.203	4.58E-03	3.77E-12	3.27E-04	206.95
11	85980958	rs7929518	G	A	0.0242	0.765	4.28E-03	1.56E-08	2.11E-04	133.69
11	112911004	rs7938812	G	T	0.0438	0.424	3.64E-03	2.71E-33	9.37E-04	593.29
12	16748721	rs11057005	G	A	-0.0209	0.43	3.58E-03	4.85E-09	2.15E-04	135.91
12	56508409	rs4759228	C	G	-0.0217	0.27	3.93E-03	3.58E-08	1.85E-04	117.39
12	69655167	rs7969559	G	A	-0.0244	0.688	3.96E-03	7.31E-10	2.55E-04	161.46
12	121389500	rs1971318	T	C	0.0285	0.141	4.93E-03	7.06E-09	1.97E-04	124.60
13	38357471	rs3904512	A	G	-0.0212	0.429	3.58E-03	3.23E-09	2.19E-04	138.83
14	29500130	rs76214862	C	A	-0.0250	0.202	4.55E-03	3.99E-08	2.01E-04	127.43
15	47935843	rs1435741	A	G	0.0294	0.425	3.59E-03	2.64E-16	4.23E-04	267.72
15	83922387	rs12441907	A	C	-0.0292	0.186	4.52E-03	1.06E-10	2.58E-04	163.48
16	717085	rs7197072	T	C	-0.0248	0.238	4.17E-03	2.77E-09	2.22E-04	140.82
16	17572674	rs4781977	C	T	-0.0239	0.205	4.36E-03	4.54E-08	1.86E-04	117.51
16	65604652	rs4785836	C	T	-0.0205	0.398	3.66E-03	2.26E-08	2.01E-04	127.09
16	87443734	rs1050847	T	C	-0.0216	0.505	3.59E-03	1.67E-09	2.34E-04	147.96
17	2072949	rs11658881	G	A	0.0201	0.418	3.61E-03	2.43E-08	1.97E-04	124.86
17	7795972	rs11078713	G	A	-0.0202	0.454	3.61E-03	2.23E-08	2.02E-04	127.68
17	30657058	rs7224742	T	C	-0.0207	0.595	3.66E-03	1.43E-08	2.07E-04	130.83
18	42632652	rs72896886	C	G	-0.0269	0.144	4.84E-03	2.75E-08	1.78E-04	112.81

18	72535282	rs11872397	A	G	-0.0248	0.252	4.09E-03	1.43E-09	2.31E-04	146.43
19	4474725	rs76608582	A	C	-0.0496	0.0389	8.26E-03	1.94E-09	1.84E-04	116.23
21	40555561	rs117143374	C	T	0.0293	0.12	5.27E-03	2.76E-08	1.81E-04	114.68
22	28781758	rs134529	C	T	-0.0200	0.349	3.66E-03	4.85E-08	1.81E-04	114.85

Table S21. Details of SNPs used for MR analysis of Coffee intake on HOA.

chr	pos	SNP	effect_allele	other_allele	beta	eaf	se	pval	R^2	F
2	27742603	rs780093	C	T	0.0133	0.616	1.66E-03	1.00E-15	8.36E-05	35.86
2	49368391	rs12989746	T	G	0.0104	0.250	1.86E-03	2.80E-08	4.02E-05	17.23
2	62780440	rs1527961	C	T	-0.0133	0.135	2.37E-03	1.70E-08	4.16E-05	17.82
4	17424930	rs2597805	T	C	0.0099	0.682	1.76E-03	2.00E-08	4.21E-05	18.05
4	106075498	rs2189234	G	T	0.0100	0.618	1.66E-03	1.80E-09	4.71E-05	20.20
5	7391462	rs12514566	A	G	-0.0114	0.337	1.71E-03	2.40E-11	5.81E-05	24.90
7	17570479	rs73075167	T	A	-0.0161	0.129	2.44E-03	5.00E-11	5.81E-05	24.90
7	32930597	rs7811609	T	C	0.0091	0.375	1.66E-03	4.00E-08	3.91E-05	16.78
7	73037956	rs34060476	G	A	0.0184	0.134	2.37E-03	7.50E-15	7.88E-05	33.78
7	75615006	rs1057868	T	C	0.0200	0.285	1.79E-03	5.40E-29	1.62E-04	69.58
8	33790200	rs78267637	G	C	-0.0254	0.038	4.32E-03	3.90E-09	4.74E-05	20.33
8	109128653	rs442355	C	G	-0.0111	0.254	1.85E-03	1.90E-09	4.71E-05	20.18
8	110443480	rs6469262	C	T	-0.0092	0.565	1.63E-03	1.90E-08	4.12E-05	17.66
10	135315795	rs117810762	A	G	0.0359	0.018	6.18E-03	6.20E-09	4.53E-05	19.42
12	11316437	rs61928609	C	A	-0.0147	0.835	2.18E-03	1.30E-11	5.97E-05	25.60
15	75174251	rs117968677	A	G	-0.0310	0.024	5.52E-03	1.90E-08	4.55E-05	19.51
16	70927078	rs8056750	T	C	0.0105	0.359	1.74E-03	1.30E-09	5.11E-05	21.90
17	60150383	rs57918684	A	G	0.0129	0.155	2.24E-03	8.60E-09	4.34E-05	18.63
18	40950954	rs630194	C	T	-0.0114	0.343	1.70E-03	2.30E-11	5.81E-05	24.93
18	55032486	rs1942965	C	T	-0.0089	0.505	1.62E-03	3.80E-08	3.96E-05	17.00

19	41353107	rs56113850	C	T	0.0127	0.578	1.63E-03	8.90E-15	7.83E-05	33.57
20	45840459	rs6063085	C	A	0.0104	0.373	1.67E-03	4.50E-10	5.07E-05	21.75
20	62891820	rs6062682	T	C	0.0104	0.465	1.64E-03	2.50E-10	5.35E-05	22.95
22	24870527	rs17842490	G	A	-0.0452	0.014	6.81E-03	3.30E-11	5.73E-05	24.58
22	41215672	rs13054099	C	T	-0.0108	0.261	1.84E-03	4.30E-09	4.48E-05	19.22

Table S22. Details of SNPs used for MR analysis of Coffee intake on KOA.

chr	pos	SNP	effect_allele	other_allele	beta	eaf	se	pval	R^2	F
2	27742603	rs780093	C	T	0.0133	0.616	1.66E-03	1.00E-15	8.36E-05	35.86
2	49368391	rs12989746	T	G	0.0104	0.250	1.86E-03	2.80E-08	4.02E-05	17.23
2	62780440	rs1527961	C	T	-0.0133	0.135	2.37E-03	1.70E-08	4.16E-05	17.82
4	17424930	rs2597805	T	C	0.0099	0.682	1.76E-03	2.00E-08	4.21E-05	18.05
4	106075498	rs2189234	G	T	0.0100	0.618	1.66E-03	1.80E-09	4.71E-05	20.20
5	7391462	rs12514566	A	G	-0.0114	0.337	1.71E-03	2.40E-11	5.81E-05	24.90
7	17570479	rs73075167	T	A	-0.0161	0.129	2.44E-03	5.00E-11	5.81E-05	24.90
7	32930597	rs7811609	T	C	0.0091	0.375	1.66E-03	4.00E-08	3.91E-05	16.78
7	73037956	rs34060476	G	A	0.0184	0.134	2.37E-03	7.50E-15	7.88E-05	33.78
7	75615006	rs1057868	T	C	0.0200	0.285	1.79E-03	5.40E-29	1.62E-04	69.58
8	33790200	rs78267637	G	C	-0.0254	0.038	4.32E-03	3.90E-09	4.74E-05	20.33
8	109128653	rs442355	C	G	-0.0111	0.254	1.85E-03	1.90E-09	4.71E-05	20.18
8	110443480	rs6469262	C	T	-0.0092	0.565	1.63E-03	1.90E-08	4.12E-05	17.66
10	135315795	rs117810762	A	G	0.0359	0.018	6.18E-03	6.20E-09	4.53E-05	19.42
12	11316437	rs61928609	C	A	-0.0147	0.835	2.18E-03	1.30E-11	5.97E-05	25.60
15	75174251	rs117968677	A	G	-0.0310	0.024	5.52E-03	1.90E-08	4.55E-05	19.51
16	70927078	rs8056750	T	C	0.0105	0.359	1.74E-03	1.30E-09	5.11E-05	21.90
17	46155786	rs62064918	T	C	-0.0103	0.245	1.88E-03	4.10E-08	3.93E-05	16.84

17	60150383	rs57918684	A	G	0.0129	0.155	2.24E-03	8.60E-09	4.34E-05	18.63
18	40950954	rs630194	C	T	-0.0114	0.343	1.70E-03	2.30E-11	5.81E-05	24.93
18	55032486	rs1942965	C	T	-0.0089	0.505	1.62E-03	3.80E-08	3.96E-05	17.00
19	41353107	rs56113850	C	T	0.0127	0.578	1.63E-03	8.90E-15	7.83E-05	33.57
20	45840459	rs6063085	C	A	0.0104	0.373	1.67E-03	4.50E-10	5.07E-05	21.75
20	62891820	rs6062682	T	C	0.0104	0.465	1.64E-03	2.50E-10	5.35E-05	22.95
22	24870527	rs17842490	G	A	-0.0452	0.014	6.81E-03	3.30E-11	5.73E-05	24.58
22	41215672	rs13054099	C	T	-0.0108	0.261	1.84E-03	4.30E-09	4.48E-05	19.22

Table S23. Details of SNPs used for MR analysis of Coffee intake on RA(M13).

chr	pos	SNP	effect_allele	other_allele	beta	eaf	se	pval	R^2	F
1	96274668	rs4615895	A	G	0.0122	0.741	1.85E-03	4.20E-11	5.72E-05	24.52
1	177855517	rs516636	A	C	0.0117	0.209	1.98E-03	4.00E-09	4.51E-05	19.33
2	637498	rs13387939	A	C	0.0166	0.828	2.14E-03	9.80E-15	7.79E-05	33.43
2	27742603	rs780093	C	T	0.0133	0.616	1.66E-03	1.00E-15	8.36E-05	35.86
2	49368391	rs12989746	T	G	0.0104	0.250	1.86E-03	2.80E-08	4.02E-05	17.23
2	62780440	rs1527961	C	T	-0.0133	0.135	2.37E-03	1.70E-08	4.16E-05	17.82
4	17424930	rs2597805	T	C	0.0099	0.682	1.76E-03	2.00E-08	4.21E-05	18.05
4	106075498	rs2189234	G	T	0.0100	0.618	1.66E-03	1.80E-09	4.71E-05	20.20
5	7391462	rs12514566	A	G	-0.0114	0.337	1.71E-03	2.40E-11	5.81E-05	24.90
5	87943710	rs13163336	A	C	0.0149	0.158	2.21E-03	1.30E-11	5.93E-05	25.44
6	51179260	rs2465037	A	C	-0.0106	0.343	1.71E-03	4.80E-10	5.09E-05	21.85
6	98312143	rs1338549	G	T	-0.0095	0.534	1.62E-03	5.60E-09	4.45E-05	19.07
6	108983527	rs9398171	T	C	0.0109	0.711	1.78E-03	1.10E-09	4.85E-05	20.79
7	17284577	rs4410790	C	T	0.0391	0.632	1.67E-03	1.20E-120	7.10E-04	304.70
7	17570479	rs73075167	T	A	-0.0161	0.129	2.44E-03	5.00E-11	5.81E-05	24.90
7	32930597	rs7811609	T	C	0.0091	0.375	1.66E-03	4.00E-08	3.91E-05	16.78

7	73037956	rs34060476	G	A	0.0184	0.134	2.37E-03	7.50E-15	7.88E-05	33.78
7	75615006	rs1057868	T	C	0.0200	0.285	1.79E-03	5.40E-29	1.62E-04	69.58
8	33790200	rs78267637	G	C	-0.0254	0.038	4.32E-03	3.90E-09	4.74E-05	20.33
8	109128653	rs442355	C	G	-0.0111	0.254	1.85E-03	1.90E-09	4.71E-05	20.18
8	110443480	rs6469262	C	T	-0.0092	0.565	1.63E-03	1.90E-08	4.12E-05	17.66
10	135315795	rs117810762	A	G	0.0359	0.018	6.18E-03	6.20E-09	4.53E-05	19.42
12	11316437	rs61928609	C	A	-0.0147	0.835	2.18E-03	1.30E-11	5.97E-05	25.60
15	75027880	rs2472297	T	C	0.0465	0.263	1.83E-03	1.10E-142	8.37E-04	359.22
15	75174251	rs117968677	A	G	-0.0310	0.024	5.52E-03	1.90E-08	4.55E-05	19.51
16	53800954	rs1421085	C	T	0.0185	0.404	1.64E-03	1.70E-29	1.66E-04	71.00
16	70927078	rs8056750	T	C	0.0105	0.359	1.74E-03	1.30E-09	5.11E-05	21.90
17	46155786	rs62064918	T	C	-0.0103	0.245	1.88E-03	4.10E-08	3.93E-05	16.84
17	60150383	rs57918684	A	G	0.0129	0.155	2.24E-03	8.60E-09	4.34E-05	18.63
18	40950954	rs630194	C	T	-0.0114	0.343	1.70E-03	2.30E-11	5.81E-05	24.93
18	55032486	rs1942965	C	T	-0.0089	0.505	1.62E-03	3.80E-08	3.96E-05	17.00
18	57852587	rs476828	C	T	0.0173	0.237	1.90E-03	5.60E-20	1.09E-04	46.73
19	18495908	rs75347775	A	G	0.0105	0.245	1.88E-03	2.70E-08	4.04E-05	17.31
19	41353107	rs56113850	C	T	0.0127	0.578	1.63E-03	8.90E-15	7.83E-05	33.57
20	45840459	rs6063085	C	A	0.0104	0.373	1.67E-03	4.50E-10	5.07E-05	21.75
20	62891820	rs6062682	T	C	0.0104	0.465	1.64E-03	2.50E-10	5.35E-05	22.95
22	24870527	rs17842490	G	A	-0.0452	0.014	6.81E-03	3.30E-11	5.73E-05	24.58
22	41215672	rs13054099	C	T	-0.0108	0.261	1.84E-03	4.30E-09	4.48E-05	19.22

Table S24. Details of SNPs used for MR analysis of Coffee intake on Seronegative RA.

chr	pos	SNP	effect_allele	other_allele	beta	eaf	se	pval	R^2	F
1	96274668	rs4615895	A	G	0.0122	0.741	1.85E-03	4.20E-11	5.72E-05	24.52

1	177855517	rs516636	A	C	0.0117	0.209	1.98E-03	4.00E-09	4.51E-05	19.33
2	637498	rs13387939	A	C	0.0166	0.828	2.14E-03	9.80E-15	7.79E-05	33.43
2	27742603	rs780093	C	T	0.0133	0.616	1.66E-03	1.00E-15	8.36E-05	35.86
2	49368391	rs12989746	T	G	0.0104	0.250	1.86E-03	2.80E-08	4.02E-05	17.23
2	62780440	rs1527961	C	T	-0.0133	0.135	2.37E-03	1.70E-08	4.16E-05	17.82
4	17424930	rs2597805	T	C	0.0099	0.682	1.76E-03	2.00E-08	4.21E-05	18.05
4	106075498	rs2189234	G	T	0.0100	0.618	1.66E-03	1.80E-09	4.71E-05	20.20
5	7391462	rs12514566	A	G	-0.0114	0.337	1.71E-03	2.40E-11	5.81E-05	24.90
5	87943710	rs13163336	A	C	0.0149	0.158	2.21E-03	1.30E-11	5.93E-05	25.44
6	51179260	rs2465037	A	C	-0.0106	0.343	1.71E-03	4.80E-10	5.09E-05	21.85
6	98312143	rs1338549	G	T	-0.0095	0.534	1.62E-03	5.60E-09	4.45E-05	19.07
6	108983527	rs9398171	T	C	0.0109	0.711	1.78E-03	1.10E-09	4.85E-05	20.79
7	17284577	rs4410790	C	T	0.0391	0.632	1.67E-03	1.20E-120	7.10E-04	304.70
7	17570479	rs73075167	T	A	-0.0161	0.129	2.44E-03	5.00E-11	5.81E-05	24.90
7	32930597	rs7811609	T	C	0.0091	0.375	1.66E-03	4.00E-08	3.91E-05	16.78
7	73037956	rs34060476	G	A	0.0184	0.134	2.37E-03	7.50E-15	7.88E-05	33.78
7	75615006	rs1057868	T	C	0.0200	0.285	1.79E-03	5.40E-29	1.62E-04	69.58
8	33790200	rs78267637	G	C	-0.0254	0.038	4.32E-03	3.90E-09	4.74E-05	20.33
8	109128653	rs442355	C	G	-0.0111	0.254	1.85E-03	1.90E-09	4.71E-05	20.18
8	110443480	rs6469262	C	T	-0.0092	0.565	1.63E-03	1.90E-08	4.12E-05	17.66
10	135315795	rs117810762	A	G	0.0359	0.018	6.18E-03	6.20E-09	4.53E-05	19.42
12	11316437	rs61928609	C	A	-0.0147	0.835	2.18E-03	1.30E-11	5.97E-05	25.60
15	75027880	rs2472297	T	C	0.0465	0.263	1.83E-03	1.10E-142	8.37E-04	359.22
15	75174251	rs117968677	A	G	-0.0310	0.024	5.52E-03	1.90E-08	4.55E-05	19.51
16	53800954	rs1421085	C	T	0.0185	0.404	1.64E-03	1.70E-29	1.66E-04	71.00
16	70927078	rs8056750	T	C	0.0105	0.359	1.74E-03	1.30E-09	5.11E-05	21.90
17	46155786	rs62064918	T	C	-0.0103	0.245	1.88E-03	4.10E-08	3.93E-05	16.84

17	60150383	rs57918684	A	G	0.0129	0.155	2.24E-03	8.60E-09	4.34E-05	18.63
18	40950954	rs630194	C	T	-0.0114	0.343	1.70E-03	2.30E-11	5.81E-05	24.93
18	55032486	rs1942965	C	T	-0.0089	0.505	1.62E-03	3.80E-08	3.96E-05	17.00
18	57852587	rs476828	C	T	0.0173	0.237	1.90E-03	5.60E-20	1.09E-04	46.73
19	18495908	rs75347775	A	G	0.0105	0.245	1.88E-03	2.70E-08	4.04E-05	17.31
19	41353107	rs56113850	C	T	0.0127	0.578	1.63E-03	8.90E-15	7.83E-05	33.57
20	45840459	rs6063085	C	A	0.0104	0.373	1.67E-03	4.50E-10	5.07E-05	21.75
20	62891820	rs6062682	T	C	0.0104	0.465	1.64E-03	2.50E-10	5.35E-05	22.95
22	24870527	rs17842490	G	A	-0.0452	0.014	6.81E-03	3.30E-11	5.73E-05	24.58
22	41215672	rs13054099	C	T	-0.0108	0.261	1.84E-03	4.30E-09	4.48E-05	19.22

Table S25. Details of SNPs used for MR analysis of Coffee intake on Seropositive RA.

chr	pos	SNP	effect_allele	other_allele	beta	eaf	se	pval	R^2	F
1	96274668	rs4615895	A	G	0.0122	0.741	1.85E-03	4.20E-11	5.72E-05	24.52
1	177855517	rs516636	A	C	0.0117	0.209	1.98E-03	4.00E-09	4.51E-05	19.33
2	637498	rs13387939	A	C	0.0166	0.828	2.14E-03	9.80E-15	7.79E-05	33.43
2	27742603	rs780093	C	T	0.0133	0.616	1.66E-03	1.00E-15	8.36E-05	35.86
2	49368391	rs12989746	T	G	0.0104	0.250	1.86E-03	2.80E-08	4.02E-05	17.23
2	62780440	rs1527961	C	T	-0.0133	0.135	2.37E-03	1.70E-08	4.16E-05	17.82
4	17424930	rs2597805	T	C	0.0099	0.682	1.76E-03	2.00E-08	4.21E-05	18.05
4	106075498	rs2189234	G	T	0.0100	0.618	1.66E-03	1.80E-09	4.71E-05	20.20
5	7391462	rs12514566	A	G	-0.0114	0.337	1.71E-03	2.40E-11	5.81E-05	24.90
5	87943710	rs13163336	A	C	0.0149	0.158	2.21E-03	1.30E-11	5.93E-05	25.44
6	51179260	rs2465037	A	C	-0.0106	0.343	1.71E-03	4.80E-10	5.09E-05	21.85
6	98312143	rs1338549	G	T	-0.0095	0.534	1.62E-03	5.60E-09	4.45E-05	19.07
6	108983527	rs9398171	T	C	0.0109	0.711	1.78E-03	1.10E-09	4.85E-05	20.79

7	17284577	rs4410790	C	T	0.0391	0.632	1.67E-03	1.20E-120	7.10E-04	304.70
7	17570479	rs73075167	T	A	-0.0161	0.129	2.44E-03	5.00E-11	5.81E-05	24.90
7	32930597	rs7811609	T	C	0.0091	0.375	1.66E-03	4.00E-08	3.91E-05	16.78
7	73037956	rs34060476	G	A	0.0184	0.134	2.37E-03	7.50E-15	7.88E-05	33.78
7	75615006	rs1057868	T	C	0.0200	0.285	1.79E-03	5.40E-29	1.62E-04	69.58
8	33790200	rs78267637	G	C	-0.0254	0.038	4.32E-03	3.90E-09	4.74E-05	20.33
8	109128653	rs442355	C	G	-0.0111	0.254	1.85E-03	1.90E-09	4.71E-05	20.18
8	110443480	rs6469262	C	T	-0.0092	0.565	1.63E-03	1.90E-08	4.12E-05	17.66
10	135315795	rs117810762	A	G	0.0359	0.018	6.18E-03	6.20E-09	4.53E-05	19.42
12	11316437	rs61928609	C	A	-0.0147	0.835	2.18E-03	1.30E-11	5.97E-05	25.60
15	75027880	rs2472297	T	C	0.0465	0.263	1.83E-03	1.10E-142	8.37E-04	359.22
15	75174251	rs117968677	A	G	-0.0310	0.024	5.52E-03	1.90E-08	4.55E-05	19.51
16	53800954	rs1421085	C	T	0.0185	0.404	1.64E-03	1.70E-29	1.66E-04	71.00
16	70927078	rs8056750	T	C	0.0105	0.359	1.74E-03	1.30E-09	5.11E-05	21.90
17	46155786	rs62064918	T	C	-0.0103	0.245	1.88E-03	4.10E-08	3.93E-05	16.84
17	60150383	rs57918684	A	G	0.0129	0.155	2.24E-03	8.60E-09	4.34E-05	18.63
18	40950954	rs630194	C	T	-0.0114	0.343	1.70E-03	2.30E-11	5.81E-05	24.93
18	55032486	rs1942965	C	T	-0.0089	0.505	1.62E-03	3.80E-08	3.96E-05	17.00
18	57852587	rs476828	C	T	0.0173	0.237	1.90E-03	5.60E-20	1.09E-04	46.73
19	18495908	rs75347775	A	G	0.0105	0.245	1.88E-03	2.70E-08	4.04E-05	17.31
19	41353107	rs56113850	C	T	0.0127	0.578	1.63E-03	8.90E-15	7.83E-05	33.57
20	45840459	rs6063085	C	A	0.0104	0.373	1.67E-03	4.50E-10	5.07E-05	21.75
20	62891820	rs6062682	T	C	0.0104	0.465	1.64E-03	2.50E-10	5.35E-05	22.95
22	24870527	rs17842490	G	A	-0.0452	0.014	6.81E-03	3.30E-11	5.73E-05	24.58
22	41215672	rs13054099	C	T	-0.0108	0.261	1.84E-03	4.30E-09	4.48E-05	19.22
